# Supplementary material for: Synthetic chimeric nucleases function for efficient genome editing
Source: Nat Commun. 2019 Dec 4;10:5524. doi: 10.1038/s41467-019-13500-y (PMC6892893; doi:10.1038/s41467-019-13500-y)
Supplement: Supplementary file 1 — Supplementary Information [file 41467_2019_13500_MOESM1_ESM.pdf]

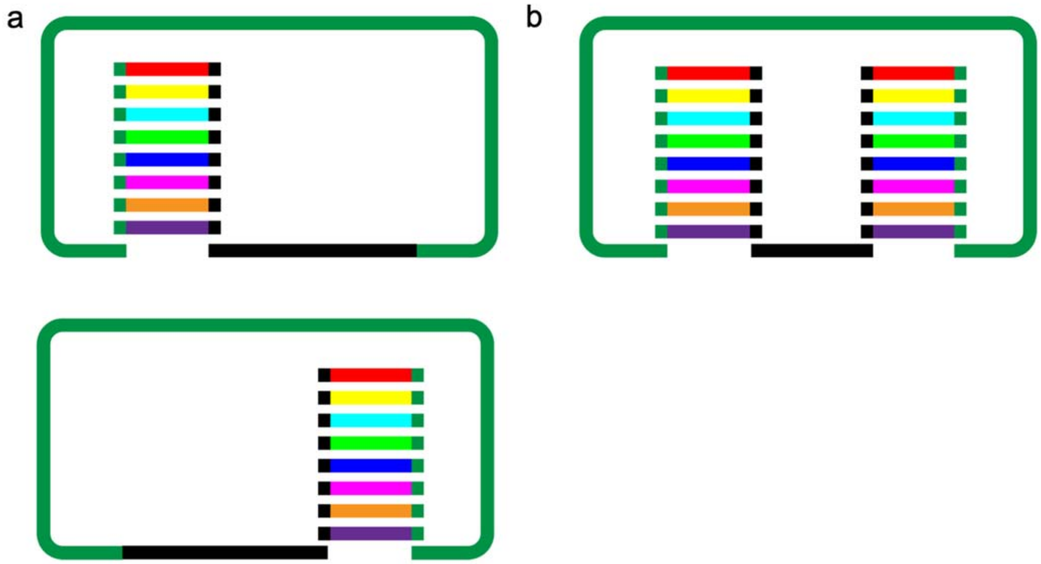

Supplementary Fig. 1 The chimera Library construction. MAD7 has the highest editing efficiency in the initial test (Fig. 1b). Thus, we used MAD7 (in black color) as template and MAD7 original plasmid (in dark green color) as backbone. Using the crossover points in Fig 1c, we designed each chimera library sequences (Table S1) using a ~40bp homology arm with MAD7 and its plasmid. The chimera sequences were shown in different colors expect black and dark green, and the homology arm were shown in black and dark green, and linked to the chimera sequences. The library construction for crossover 1 (a) and 2 (b) were used Gibson assembly method. (a) The library construction used 1 crossover. The library variants for 1 crossover should be 48 (8 chimera sequences x 6 positions) theoretically. (b) The library construction used 2 crossovers. The library variants for 2 crossovers should be 512 (8 chimera sequences x 8 chimera sequences x 8 combinations) theoretically. Thus, the total number of variants should be 560 theoretically.

CLUSTAL O(1.2.4) multiple sequence alignment

```
CR_Cas12a      --------MSFDSFTNLYSLSKTLKFEMRPVGNTQKMLDN------AGVFEKDKLIQKKY     46
SD_Cas12a      -------MSSLTKFTNKYSKQLTIKNELIPVGKTLENIKE------NGLIDGDEQLNENY     47
As_Cas12a      -------MTQFEGFTNLYQVSKTLRFELIPQGKTLKHIQE------QGFIEEDKARNDHY     47
TX_Cas12a      -----MTKTFDSEFFNLYSLQKTVRFELKPVGETASFVEDFKNEGLKRVVSEDERRAVDY     55
Lb_Cas12a      -------MSKLEKFTNCYSLSKTLRFKAIPVGKTQENIDN------KRLLVEDEKRAEDY     47
PC_Cas12a      -------MDSLKDFTNLYPVSKTLRFELKPVGKTLENIEK------AGILKEDEHRAESY     47
FB_Cas12a      ---------MTNKFTNQYSLSKTLRFELIPQGKTLEFIQE------KGLLSQDKQRAESY     45
MAD7           MN---NGTNNFQNFIGISSLQKTLRNALIPTETTQQFIVK------NGIIKEDELRGENR     51
CT_Cas12a      -------MNNYDEFTKLYPIQKTIRFELKPQGRTMEHLET------FNFFEEDRDRAEKY     47
CA_Cas12a      MHTGGLLSMDAKEFTGQYPLSKTLRFELRPIGRTWDNLEA------SGYLAEDRHRAECY     54
                            *      . *::    *   * . :           .  *.

CR_Cas12a      GKTKPYFDRLHREFIEEALTGVELIGLDENFR----TLV----DW--QKDKKNNVAM-KA     95
SD_Cas12a      QKAKIIVDDFLRDFINKALNNTQIGN--------WRELADALNKEDEDNIEKLQDKI-RG     98
As_Cas12a      KELKPIIDRIYKTYADQCLQLVQL-D--------WENLSAAIDSYRKEKTEETRN----A     94
TX_Cas12a      QKVKEIIDDYHRDFIEESLNYFPEQVSKD----ALEQAFHLYQKLKAAKVEEREKAL-KE     110
Lb_Cas12a      KGVKKLLDRYYLSFINDVLHSIKLK--------NLNNYISLFRKK--TRTEKENKEL-EN     96
PC_Cas12a      RRVKKIIDTYHKVFIDSSLENMAKMGIENEIKAMLQSFCELYKKD--HRTEGEDKAL-DK     104
FB_Cas12a      QEMKKTIDKFHKYFIDLALSNAKLT--------HLETYLELYNKS--AETKKEQKFK-DD     94
MAD7           QILKDIMDDYYRGFISETLSSIDDID--------WTSLFEKMEIQ--LK-NGDNKD---T     97
CT_Cas12a      KILKEAIDEYHKKFIDEHLTNM-SLD--------WNSLKQISEKY--YK-SREEKDKK-V     94
CA_Cas12a      PRAKELLDDNHRAFLNRVLPQI-DMD--------WHPIAEAFCKV--HK-NPGNKELAQD     102
                  *  .*     : .  *                             . .

CR_Cas12a      YENSLQRLRTEIGKIFNLKAEDWVK------NKYPILGLKNKNTDILFEEAVFGILKARY     149
SD_Cas12a      IIVSKFETFDLFSSYSIKKDEKIIDDDNDVEEEELDLGKKTSSFKYIFKKNLFKLVLPSY     158
As_Cas12a      LIEEQATYRNAIHDYFIGRTDNLTDAINK---------RHAEIYKGLFKAELFNGKVLKQ     145
TX_Cas12a      WEALQKKLREKVVKCFSDSNKA--------------------RFSRIDKKELIKEDLINW     150
Lb_Cas12a      LE---INLRKEIAKAFKGNE----------------------GYKSLFKKDIIETILPEF     131
PC_Cas12a      IR---AVLRGLIVGAFTGVCGRREN------------TVQNEKYESLFKEKLIKEILPDF     149
FB_Cas12a      LKKVQDNLRKEIVKSFSDGDAKS-------------------IFAILDKKELITVELEKW     135
MAD7           LIKEQTEYRKAIHKKFANDD----------------------RFKNMFSAKLISDILPEF     135
CT_Cas12a      FLSEQKRMRQEIVSEFKKDD----------------------RFKDLFSKKLFSELLKEE     132
CA_Cas12a      YNLQLSKRRKEISAYLQDAD----------------------GYKGLFAKPALDEAMKI-     139
                          .                                  :     :

CR_Cas12a      GEEKDTFIEVEEIDKTGKSKINQISIFDSWKGFTGYFKKFFETRKNFYKNDGTSTAIATR     209
SD_Cas12a      -------L--KTT------NQDKLKIISSFDNFSTYFRGFFENRKNIFTKKPISTSIAYR     203
As_Cas12a      -------LGTVTT------TEHENALLRSFDKFTTYFSGFYENRKNVFSAEDISTAIPHR     192
TX_Cas12a      -------LVAQNRED-------DIPTVETFNNFTTYFTGFHENRKNIYSKDDHATAISFR     196
Lb_Cas12a      -------LDDKDE----------IALVNSFNGFTTAFTGFFDNRENMFSEEAKSTSIAFR     174
PC_Cas12a      -------VLSTEAESLPFSVEEATRSLKEFDSFTSYFAGFYENRKNIYSTKPQSTAIAYR     202
FB_Cas12a      -------FENNEQKD--------IYFDEKFKTFTTYFTGFHQNRKNMYSVEPNSTAIAYR     180
MAD7           -------VIHNNNYS-ASEKEEKTQVIKLFSRFATSFKDYFKNRANCFSADDISSSSCHR     187
CT_Cas12a      -------IYKKGN-------HQEIDALKSFDKFSGYFIGLHENRKNMYSDGDEITAISNR     178
CA_Cas12a      -------AKENGN-------ESDIEVLEAFNGFSVYFTGYHESRENIYSDEDMV-SVAYR     184
                                            :. *:  *   ...* * :.      :   *

CR_Cas12a      IIDQNLKRFIDNLSIVESVRQKVDLA----ET--------EKSFSISLSQFFSIDFYNKC     257
SD_Cas12a      IVHDNFPKFLDNIRCFNVWQTECPQL--IVKADNYLKSKNVIAKDKSLANYFTVGAYDYF     261
As_Cas12a      IVQDNFPKFKENCHIFTRLITAVPSL--REHFENVKKAIG-IFVSTSIEEVFSFPFYNQL     249
TX_Cas12a      LIHENLPKFFDNVISFNKLKEGFPEL-KFDKV------KEDLEVDYDLKHAFEIEYFVNF     249
Lb_Cas12a      CINENLTRYISNMDIFEKVDAIFDKH-EVQEI-----KEKILNSDYDVEDFFEGEFFNFV     228
PC_Cas12a      LIHENLPKFIDNILVFQKIKEPIA--KELEHIRADFSAGGYIKKDERLEDIFSLNYYIHV     260
FB_Cas12a      LIHENLPKFLENAKAFEKIKQVESLQVNFRELMGEFGDEGLIFV-NELEEMFQINYYNDV     239
MAD7           IVNDNAEIFFSNALVYRRIVKSLSND-DINKISGDM---KDSLKEMSLEEIYSYEKYGEF     243
CT_Cas12a      IVNENFPKFLDNLQKYQEARKKYPE--WIIKAESAL-----VAHNIKMDEVFSLEYFNKV     231
CA_Cas12a      ITEDNFPRFVSNALIFDKLNESHPD--IISEVSGNL-----GVD--DIGKYFDVSNYNNF     235
                 .:*   : .*                  .                : . :    :

CR_Cas12a      LLQDGIDYYNKIIGGETLKNG-EKLIGLNELINQYRQNNK-------DQKIPFFKLLDKQ     309
SD_Cas12a      LSQNGIDFYNNIIGGLPAFAGHEKIQGLNEFINQECQKDSELKSKLKNRHAFKMAVLFKQ     321
As_Cas12a      LTQTQIDLYNQLLGGISREAGTEKIKGLNEVLNLAIQKNDETAHII-ASLPHRFIPLFKQ     308
TX_Cas12a      VTQAGIDQYNYLLGGKTLEDG-TKKQGMNEQINLFKQQQTRDK----ARQIPKLIPLFKQ     304
Lb_Cas12a      LTQEGIDVYNAIIGGFVTESG-EKIKGLNEYINLYNQK--T------KQKLPKFKPLYKQ     279
PC_Cas12a      LSQAGIEKYNALIGKIVTEGD-GEMKGLNEHINLYNQQ--RGR----EDRLPLFRPLYKQ     313
```

```
FB_Cas12a      LSQNGITIYNSIISGF-TKND-IKYKGLNEYINNYNQT--KDK----KDRLPKLKQLYKQ     291
MAD7           ITQEGISFYNDICGKV------------NSFMNLYCQKNK--E----NKNLYKLQKLHKQ     285
CT_Cas12a      LNQEGIQRYNLALGGYVTKSG-EKMMGLNDALNLAHQSEK--S----SKGRIHMTPLFKQ     284
CA_Cas12a      LSQAGIDDYNHIIGGHTTEDG-L-IQAFNVVLNLRHQKD---P----GFEKIQFKQLYKQ     286
               : *  *  **   .              *  :*   *                :  * **  Crossover 1

CR_Cas12a      ILSEK----ILFLDEIKNDTELIEALSQFAKTAEEK---------TKIVKKLFA--DFVE     354
SD_Cas12a      ILSDR-E-KSFVIDEFESDAQVIDAVKNFYAEQCKD---------NNVIFNLLNLIKNIA     370
As_Cas12a      ILSDR-NTLSFILEEFKSDEEVIQSFCKYKTLL-RN---------ENVLETAEAL---FN     354
TX_Cas12a      ILSERTESQSFIPKQFESDQELFDSLQKLHNNC------------QDKFTVLQQA---IL     349
Lb_Cas12a      VLSDR-ESLSFYGEGYTSDEEVLEVFRNTLNKNS---------EIFSSIKKLEKL---FK     326
PC_Cas12a      ILSDR-EQLSYLPESFEKDEELLRALKEFYDHIA-----------EDILGRTQQL---MT     358
FB_Cas12a      ILSDR-ISLSFLPDAFTDGKQVLKAIFDFYKINLLSYTIEGQEESQNLLLLIRQT---IE     347
MAD7           ILCIA-DTSYEVPYKFESDEEVYQSVNGFLDNISSK----------HIVERLRKI---GD     331
CT_Cas12a      ILSEK-ESFSYIPDVFTEDSQLLPSIGGFFAQIEND-------KDGNIFDRALEL---IS     333
CA_Cas12a      ILSVR-TSKSYIPKQFDNSKEMVDCICDYVSKIEKS----------ETVERALKL---VR     332
               :*.              .. ::   .                      .

CR_Cas12a      NNSKYDLAQIYISQ-EAFNTISNKWTSETETFAKYLFEAMKSGKL-------AKYEKKDN     406
SD_Cas12a      FLSDDELDGIFIEG-KYLSSVSQKLYSDWSKLRNDIEDSANSKQGNKELAKKIKTNK--G     427
As_Cas12a      ELNSIDLTHIFISH-KKLETISSALCDHWDTLRNALYERRISELTGKI----TKSAK--E     407
TX_Cas12a      GLAEADLKKVFIKT-SDLNALSNTIFGNYSVFSDALNLYKESLKTKKAQ-----------     397
Lb_Cas12a      NFDEYSSAGIFVKNGPAISTISKDIFGEWNVIRDKWNAEYDDIHLKKKAVVTEKYEDDRR     386
PC_Cas12a      SISEYDLSRIYVRNDSQLTDISKKMLGDWNAIYMARERAYDHEQAPK--RITAKYERDRI     416
FB_Cas12a      NLSSFDTQKIYLKNDTHLTTISQQVFGDFSVFSTALNYWYETKVNPK--FET-EYSKANE     404
MAD7           NYNGYNLDKIYIVS-KFYESVSQKTYRDWETINTALEIHYNNILPGNGKSKADK------     384
CT_Cas12a      SYAEYDTERIYIRQ-ADINRVSNVIFGEWGTLGGLMREYKADSINDINLERT--------     384
CA_Cas12a      NISSFDLRGIFVNK-KNLRILSNKLIGDWDAIETALMHSSSSEND---------------     376
                    .   :::        :*.    .   :

CR_Cas12a      ----SY-KFP---------DFIALSQMKSALLSISLEGHFWKEKYYKISKFQ-EKTNWEQ     451
SD_Cas12a      ----DVEKAISK-------YEFSLSELNSIVHDNT-------------------------     451
As_Cas12a      ----KVQRSLKH-------EDINLQEIISAA--GK-------------------------     429
TX_Cas12a      ---EAFEKLPAH-------SIHDLIQYLEQFNSSL-----------DAEKQQSTDTVLNY     436
Lb_Cas12a      ---KSFKKIGSF-------SLEQLQEYADADLSV-----------------------VEK     413
PC_Cas12a      ---KALKGEESISLANLNSCIAFLDNV--------------------------RDCRVDT     447
FB_Cas12a      KKREILDKAKAVFTKQDYFSIAFLQEVLSEYILTLDH-------TSDIVKKHSSNCIADY     457
MAD7           -----VKKAVKN---DLQKSITEINELVSNYKLCS------------------DD---NI     415
CT_Cas12a      -----CKKVDKW---L-DSKEFALSDVLEAIKRTG------------------NNDAFN-     416
CA_Cas12a      -------KKSVY---D-SAEAFTLDDIFSSVKKFS------------------DASAEDI     407
                                      : :

CR_Cas12a      FLAIFLY---EFNSLFSDKINTKDGETKQVGYYLFAKDLHNLIL-------SEQI-----     496
SD_Cas12a      ----------KFSDLLSCTLHKVASE--K-----LVKV--------------NEGDWPKH     480
As_Cas12a      ----------ELSEAFKQKTSEILS---H-----AHAA--------------LDQPLPTT     457
TX_Cas12a      FIKT--------DELYSRFIKSTSEAFTQ-----VQPLFELEALSSKRRPPESEDEGAKG     483
Lb_Cas12a      L-----------KEIIIQKVDEIYKVYGS-----SEKLFDADF------V--LEKSL---     446
PC_Cas12a      YLSTLGQK--EGPHGLSNLVENVFASYHE-----AEQLLSFPY------P--EENNL---     489
FB_Cas12a      FKNHFVAKKENETDKTFDFIANITAKYQC-----IQGILENAD------Q--YEDEL---     501
MAD7           ----------K-AETYIHEISHILNNFEA-------QELK--Y------NPEI-----HL     444
CT_Cas12a      --------------EYISKMRTAREKIDA-----ARKEMK--F------I-SE-----KI     443
CA_Cas12a      ----------GNRAEDICRVISETAPFIN-----DLRAVD--L------D-SL-----ND     438

CR_Cas12a      DIPKDSKVTIKDFADSVLTIYQMAKYFAVEKKRAWLAEYELD-SFYTQPDTGYLQFYDNA     555
SD_Cas12a      LKNNEEKQKIKEPLDALLEIYNTLLIFNCKS------FNKN-GNFYVDYD----RCINEL     529
As_Cas12a      LKKQEEKEILKSQLDSLLGLYHLLDWFAVDE------SNEVDPEFSARLT----GIKLEM     507
TX_Cas12a      QEGFEQIKRIKAYLDTLMEAVHFAKPLYLVKGRKMIEGLDKDQSFYEAFE----MAYQEL     539
Lb_Cas12a      KKNDAVVAIMKDLLDSVKSFENYIKAFFGEGK-----ETNRDESFYGDFV----LAYDIL     497
PC_Cas12a      IQDKDNVVLIKNLLDNISDLQRFLKPLWGMGD-----EPDKDERFYGEYN----YIRGAL     540
FB_Cas12a      KQDQKLIDNLKFFLDAILELLHFIKPLHLKSES----ITEKDTAFYDVFE----NYYEAL     553
MAD7           VESELKASELKNVLDVIMNAFHWCSVFMTE-E-----LVDKDNNFYAELE----EIYDEI     494
CT_Cas12a      SGDEESIHIIKTLLDSVQQFLHFFNLFKAR-Q-----DIPLDGAFYAEFD----EVHSKL     493
CA_Cas12a      DGYEAAVSKIRESLEPYMDLFHELEIFSVG-D-----EFPKCAAFYSELE----EVSEQL     488
                        ::   :      .    :                 *

CR_Cas12a      YEDIVQVYNKLRNYLTKKPYSEEKWKLNFENSTLANGWDKNKESDNSAVILQKGGKYYLG     615
SD_Cas12a      -SSVVYLYNKTRNYCTKKPYNTDKFKLNFNSPQLGEGFSKSKENDCLTLLFKKDDNYYVG     588
As_Cas12a      -EPSLSFYNKARNYATKKPYSVEKFKLNFQMPTLASGWDVNKEKNNGAILFVKNGLYYLG     566
```

```
TX_Cas12a       ESLIIPIYNKARSYLSRKPFKADKFKINFDNNTLLSGWDANKETANASILFKKDGLYYLG     599
Lb_Cas12a       -LKVDHIYDAIRNYVTQKPYSKDKFKLYFQNPQFMGGWDKDKETDYRATILRYGSKYYLA     556
PC_Cas12a       -DQVIPLYNKVRNYLTRKPYSTRKVKLNFGNSQLLSGWDRNKEKDNSCVILRKGQNFYLA     599
FB_Cas12a       -SLLTPLYNMVRNYVTQKPYSTEKIKLNFENAQLLNGWDANKEGDYLTTILKKDGNYFLA     612
MAD7            -YPVISLYNLVRNYVTQKPYSTKKIKLNFGIPTLADGWSKSKEYSNNAIILMRDNLYYLG     553
CT_Cas12a       -FAIVPLYNKVRNYLTKNNLNTKKIKLNFKNPTLANGWDQNKVYDYASLIFLRDGNYYLG     552
CA_Cas12a       -IEIIPLFNKARSFCTRKRYSTDKIKVNLKFPTLADGWDLNKERDNKAAILRKDGKYYLA     547
                      .::  *.: :::  .  * *: :    :  *:. .*       ::  .  :::.
```

Crossover 2

```
CR_Cas12a       LITKGHNKIFDDRFQE---------------KFIVGIEGGKYEKIVYKFFPDQAKMFPKV     660
SD_Cas12a       IIRKGAKINFDDT------------------QAIADNTDNCIFKMNYFLLKDAKKFIPKC     630
As_Cas12a       IMPKQKGRYKALS------------------FEPTEKTSEGFDKMYYDYFPDAAKMIPKC     608
TX_Cas12a       IMPKGKTFLFDYFVSSEDSEKLKQRRQKTAEEALAQDGESYFEKIRYKLLPGASKMLPKV     659
Lb_Cas12a       IMDKKYAKCLQKI--------------------DKDDVNGNYEKINYKLLPGPNKMLPKV     596
PC_Cas12a       IMNNRHKRSFENKVL-----------------PEYKEGEPYFEKMDYKFLPDPNKMLPKV     642
FB_Cas12a       IMDKKHNKAF----------------------QKFPEGKENYEKMVYKLLPGVNKMLPKV     650
MAD7            IFNAKNKPDKKII------------------EGNTSENKGDYKKMIYNLLPGPNKMIPKV     595
CT_Cas12a       IINPKRKKNIKFE------------------Q--GSGNGPFYRKMVYKQIPGPNKNLPRV     592
CA_Cas12a       ILDMKKDLSS-IR------------------T--SDEDESSFEKMEYKLLPSPVKMLPKI     586
                ::                                         *: *  : .  * :*:

CR_Cas12a       CFS-AKGLEFFRPSEEILRIYNN-----------------AEFKKG--------------     688
SD_Cas12a       SIQLKEVKAHFKKSEDDYILSDKEKFASPLVIKKSTFLLATAHVKGKKGNIKKFQKEYSK     690
As_Cas12a       STQLKAVTAHFQTHTTPILLSN--NFIEPLEITKEIYDLNNP-----EKEPKKFQTAYAK     661
TX_Cas12a       FFS-NKNIGFYNPSDDILRIRNT-----------------ASHTKNGT--PQ---KGHS-     695
Lb_Cas12a       FFS-KKWMAYYNPSEDIQKIYKN-----------------GTFKKG--------------     624
PC_Cas12a       FLS-KKGIEIYKPSPKLLEQYGH-----------------GTHKKG--------------     670
FB_Cas12a       FFS-NKNIAYFNPSKELLENYKK-----------------ETHKKG--------------     678
MAD7            FLSSKTGVETYKPSAYILEGYKQ-----------------NKHIKSS-------------     625
CT_Cas12a       FLTSTKGKKEYKPSKEIIEGYEA-----------------DKHIRG--------------     621
CA_Cas12a       FVKSKAAKEKYGLTDRMLECYDK-----------------GMHKSG--------------     615
                          :

CR_Cas12a       ----ETYSIDSMQKLIDFYKDCLTKYEGWA-CYTFRHLKPTEEYQNNIGEFFRDVAEDGY     743
SD_Cas12a       E--NPTEYRNSLNEWIAFCKEFLKTYKAAT-IFDITTLKKAEEYA-DIVEFYKDVDNLCY     746
As_Cas12a       KTGDQKGYREALCKWIDFTRDFLSKYTKTT-SIDLSSLRPSSQYK-DLGEYYAELNPLLY     719
TX_Cas12a       ---KVEFNLNDCHKMIDFFKSSIQKHPEWG-SFGF-TFSDTSDFE-DMSAFYREVENQGY     749
Lb_Cas12a       ----DMFNLNDCHKLIDFFKDSISRYPKWSNAYDF-NFSETEKYK-DIAGFYREVEEQGY     678
PC_Cas12a       ----DTFSMDDLHELIDFFKHSIEAHEDWK-QFGF-KFSDTATYE-NVSSFYREVEDQGY     723
FB_Cas12a       ----DTFNLEHCHTLIDFFKDSLNKHEDWK-YFDF-QFSETKSYQ-DLSGFYREVEHQGY     731
MAD7            ----KDFDITFCHDLIDYFKNCIAIHPEWK-NFGF-DFSDTSTYE-DISGFYREVELQGY     678
CT_Cas12a       ----DKFDLDFCHKLIDFFKESIEKHKDWS-KFNF-YFSPTESYG-DISEFYLDVEKQGY     674
CA_Cas12a       ----SAFDLGFCHELIDYYKRCIAEYPGWD-VFDF-KFRETSDYG-SMKEFNEDVAGAGY     668
                               * : :  :  :        :  :  :  :  .:  :  ::    *

CR_Cas12a       RIDFQGISDQYIHEKNEKGELHLFEIHNKDWNLDKARDGKSKTTQKNLHTLYFESLFSND     803
SD_Cas12a       KLEFCPIKTSFIENLIDNGDLYLFRINNKDFSS-------KSTGTKNLHTLYLQAIFDER     799
As_Cas12a       HISFQRIAEKEIMDAVETGKLYLFQIYNKDFAK-------GHHGKPNLHTLYWTGLFSPE     772
TX_Cas12a       VISFDKIKETYIQSQVEQGNLYLFQIYNKDFSP-------YSKGKPNLHTLYWKALFEEA     802
Lb_Cas12a       KVSFESASKKEVDKLVEEGKLYMFQIYNKDFSD-------KSHGTPNLHTMYFKLLFDEN     731
PC_Cas12a       KLSFRKVSESYVYSLIDQGKLYLFQIYNKDFSP-------CSKGTPNLHTLYWRMLFDER     776
FB_Cas12a       KINFKNIDSEYIDGLVNEGKLFLFQIYSKDFSP-------FSKGKPNMHTLYWKALFEEQ     784
MAD7            KIDWTYISEKDIDLLQEKGQLYLFQIYNKDFSK-------KSTGNDNLHTMYLKNLFSEE     731
CT_Cas12a       RMHFENISAETIDEYVEKGDLFLFQIYNKDFVK-------AATGKKDMHTIYWNAAFSPE     727
CA_Cas12a       YMSLRKIPCSEVYRLLDEKSIYLFQIYNKDYSE-------NAHGNKNMHTMYWEGLFSPQ     721
                 :         :    :  .:.:*.* .**:               ::**:*    *.
```

Crossover 3 4

```
CR_Cas12a       NVVQNFPIKLNGQAEIFYRPKTEKDKLE--S-----------------------------     832
SD_Cas12a       NL-NNPTIMLNGGAELFYRKESIEQKNRITHKAGSILVNKVCK-D-----------GTSL     846
As_Cas12a       NL-AKTSIKLNGQAELFYRPKSRM--KRMAHRLGEKMLNKKLKDQ-----------KTPI     818
TX_Cas12a       NL-NNVVAKLNGEAEIFFRRHSIKASDKVVHPANQAIDNKNPHTE---------------     846
Lb_Cas12a       NH-GQ--IRLSGGAELFMRRASLKKEELVVHPANSPIANKNPDNP---------------     773
PC_Cas12a       NL-ADVIYKLDGKAEIFFREKSLKNDHPTHPAGK-PIK------KKSR------------     816
FB_Cas12a       NL-QNVIYKLNGQAEIFFRKASIKPKNIILHKKKIKIAKKHFIDKKTKT-------SEIV     836
MAD7            NL-KDIVLKLNGEAEIFFRKSSIK--NPIIHKKGSILVNRTYEAEEKDQFGNIQIVRKNI     788
CT_Cas12a       NL-QDVVVKLNGEAELFYRDKSDI--KEIVHREGEILVNRTYNG------------RTPV     772
CA_Cas12a       NL-ESPVFKLSGGAELFFRKSSIPNDAKTVHPKGSVLVPRNDVN------------GRRI     768
                *   .    *.* **:* *  :
```

```
CR_Cas12a      -----------------------------------KKDKKGNKVIDHKRYSENKIFFHVP      857
SD_Cas12a      DDKIRNEIYQYENK--FIDTLSDEAKKVLPN---VIKKEATHDITKDKRFTSDKFFFHCP      901
As_Cas12a      PDTLYQELYDYVNHR-LSHDLSDEARALLPN---VITKEVSHEIIKDRRFTSDKFFFHVP      874
TX_Cas12a      ----------------------------------KTQSTFEYDLVKDKRYTQDKFFFHVP      872
Lb_Cas12a      ----------------------------------KKTTTLSYDVYKDKRFSEDQYELHIP      799
PC_Cas12a      -------------QK-------------------KGEESLFEYDLVKDRHYTMDKFQFHVP      845
FB_Cas12a      PVQTIKNLNMYYQGKISEKELTQDDLRYIDNFSIFNEKNKTIDIIKDKRFTVDKFQFHVP      896
MAD7           PENIYQELYKYFNDK-SDKELSDEAAKLKNV---VGHHEAATNIVKDYRYTYDKYFLHMP      844
CT_Cas12a      PDKIHKKLTDYHN-G-RTKD-LGEAKEYLDK---VRYFKAHYDITKDRRYLNDKIYFHVP      826
CA_Cas12a      PDSIYRELTRYFNRG-DCRI-SDEAKSYLDK---VKTKKADHDIVKDRRFTVDKMMFHVP      823
                                                         .: .. ::  ::  :* *
                                                                         Crossover 5

CR_Cas12a      LTLNRTKND-SYRFNAQINNFLANNKDINIIGVDRGEKHLVYYSVITQASDILESGSLNE      916
SD_Cas12a      LTINYKEGD-TKQFNNEVLSFLRGNPDINIIGIDRGERNLIYVTVINQKGEILDSVSFNT      960
As_Cas12a      ITLNYQAANSPSKFNQRVNAYLKEHPETPIIGIDRGERNLIYITVIDSTGKILEQRSLNT      934
TX_Cas12a      ISLNFKAQG-VSKFNDKVNGFLKGNPDVNIIGIDRGERHLLYFTVVNQKGEILVQESLNT      931
Lb_Cas12a      IAINKCPKN-IFKINTEVRVLLKHDDNPYVIGIDRGERNLLYIVVVDGKGNIVEQYSLNE      858
PC_Cas12a      ITMNFKCSA-GSKVNDMVNAHIREAKDMHVIGIDRGERNLLYICVIDSRGTILDQISLNT      904
FB_Cas12a      ITMNFKATG-GSYINQTVLEYLQNNPEVKIIGLDRGERHLVYLTLIDQQGNILKQESLNT      955
MAD7           ITINFKANK-TGFINDRILQYIAKEKDLHVIGIDRGERNLIYVSVIDTCGNIVEQKSFNI      903
CT_Cas12a      LTLNFKANG-KKNLNKMVIEKFLSDEKAHIIGIDRGERNLLYYSIIDRSGKIIDQQSLNV      885
CA_Cas12a      IAMNFKAIS-KPNLNKKVIDGIIDDQDLKIIGIDRGERNLIYVTMVDRKGNILYQDSLNI      882
               :::*         .*  :   :    .  :**:****::*:*  ::   . *: . *:*

CR_Cas12a      L----NG----VNYAEKLGKKAENREQARRDWQDVQGIKDLKKGYISQVVRKLADLAIKH      968
SD_Cas12a      VTNKSSKIEQTVDYEEKLAVREKERIEAKRSWDSISKIATLKEGYLSAIVHEICLLMIKH      1020
As_Cas12a      I--------QQFDYQKKLDNREKERVAARQAWSVVGTIKDLKQGYLSQVIHEIVDLMIHY      986
TX_Cas12a      LMSD---KGHVNDYQQKLDKKEQERDAARKSWTTVENIKELKEGYLSHVVHKLAHLIIKY      988
Lb_Cas12a      IINNFNGIRIKTDYHSLLDKKEKERFEARQNWTSIENIKELKAGYISQVVHKICELVEKY      918
PC_Cas12a      I----ND----IDYHDLLESRDKDRQQERRNWQTIEGIKELKQGYLSQAVHRIAELMVAY      956
FB_Cas12a      I----TDSKISTPYHKLLDNKENERDLARKNWGTVENIKELKEGYISQVVHKIATLMLEE      1011
MAD7           V----NG----YDYQIKLKQQEGARQIARKEWKEIGKIKEIKEGYLSLVIHEISKMVIKY      955
CT_Cas12a      I----DG----FDYREKLNQREIEMKDARQSWNAIGKIKDLKEGYLSKAVHEITKMAIQY      937
CA_Cas12a      L----NG----YDYRKALDVREYDNKEARRNWTKVEGIRKMKEGYLSLAVSKLADMIIEN      934
               :            *   *  :       :: *  :  *  :* **:*  :  .:  :

CR_Cas12a      NAIIILEDLNMRFKQVRGG-IEKSIYQQLEKALIDKLSFLVDKGEKNPEQAGHLLKAYQL      1027
SD_Cas12a      NAIVVLENLNAGFKRIRGGLSEKSVYQKFEKMLINKLNYFVSKKESDWNKPSGLLNGLQL      1080
As_Cas12a      QAVVVLENLNFGFKSKRTGIAEKAVYQQFEKMLIDKLNCLVLKD-YPAEKVGGVLNPYQL      1045
TX_Cas12a      NAIVCLEDLNFGFKRGRFK-VEKQVYQKFEKALIDKLNYLVFKE-KELGEVGHYLTAYQL      1046
Lb_Cas12a      DAVIALEDLNSGFKNSRVK-VEKQVYQKFEKMLIDKLNYMVDKK-SNPCATGGALKGYQI      976
PC_Cas12a      KAVVALEDLNMGFKRGRQK-VESSVYQQFEKQLIDKLNYLVDKK-KRPEDIGGLLRAYQF      1014
FB_Cas12a      NAIVVMEDLNFGFKRGRFK-VEKQIYQKLEKMLIDKLNYLVLKD-KQPQELGGLYNALQL      1069
MAD7           NAIIAMEDLSYGFKKGRFK-VERQVYQKFETMLINKLNYLVFKD-ISITENGGLLKGYQL      1013
CT_Cas12a      NAIVVMEELNYGFKRGRFK-VEKQIYQKFENMLIDKMNYLVFKD-APDESPGGVLNAYQL      995
CA_Cas12a      NAIIVMEDLNHGFKAGRSK-IEKQVYQKFESMLINKLGYMVLKD-KSIDQSGGALHGYQL      992
               .*:: :*:*.  **  *    *  :**::*. **:*:. :* *         .      *:

CR_Cas12a      SAPFETFQKMGKQTGIIFYTQASYTSKSDPVTGWRPHLYLKY-FSAKKAKDDIAKFTKIE      1086
SD_Cas12a      SDQFESFEKLGIQSGFIFYVPAAYTSKIDPTTGFANVLNLSKVRNVDAIKSFFSNFNEIS      1140
As_Cas12a      TDQFTSFAKMGTQSGFLFYVPAPYTSKIDPLTGFVDPFVWKTIKNHESRKHFLEGFDFLH      1105
TX_Cas12a      TAPFESFKKLGKQSGILFYVPADYTSKIDPTTGFVNFLDLRY-QSVEKAKQLLSDFNAIR      1105
Lb_Cas12a      TNKFESFKSMSTQNGFIFYIPAWLTSKIDPSTGFVNLLKTKY-TSIADSKKFISSFDRIM      1035
PC_Cas12a      TAPFKSFKEMGKQNGFLFYIPAWNTSNIDPTTGFVNLFHAQY-ENVDKAKSFFQKFDSIS      1073
FB_Cas12a      TNKFESFQKMGKQSGFLFYVPAWNTSKIDPTTGFVNYFYTKY-ENVDKAKAFFEKFEAIR      1128
MAD7           TYIPDKLKNVGHQCGCIFYVPAAYTSKIDPTTGFVNIFKFKDLT-VDAKREFIKKFDSIR      1072
CT_Cas12a      TNPLESFAKLGKQTGILFYVPAAYTSKIDPTTGFVNLFNTSSKTNAQERKEFLQKFESIS      1055
CA_Cas12a      ANHVTTLASVGKQCGVIFYIPAAFTSKIDPTTGFADLFALSNVKNVASMREFFSKMKSVI      1052
               :    .: .:. * * :**  *  **: ** **:   :           :  :  :  :
                                        Crossover 6

CR_Cas12a      FVND---RFELTY-DIKDFQQAKE-----YPNKTVWKVC---------------SNVERF      1122
SD_Cas12a      YSKK-EALFKFSFDLDS--LSKKGFSSFVKFSKSKWNVY---------------TFGERI      1182
As_Cas12a      YDVK-TGDFILHFKMNRNLSFQRGLPGFM----PAWDIVFEKNETQFDAKGTPFIAGKRI      1160
TX_Cas12a      FNSV-QNYFEFEI-DYKKLTPKRK-----VGTQSKWVIC---------------TYGDVR      1143
Lb_Cas12a      YVPE-EDLFEFAL-DYKNFSR-TD-----ADYIKKWKLY---------------SYGNRI      1072
PC_Cas12a      YNPK-KDWFEFAF-DYKNFTK-KA-----EGSRSMWILC---------------THGSRI      1110
FB_Cas12a      FNAE-KKYFEFEVKKYSDFNP-KA-----EGTQQAWTIC---------------TYGERI      1166
MAD7           YDSE-KNLFCFTF-DYNNFITQNT-----VMSKSSWSVY---------------TYGVRI      1110
CT_Cas12a      YSAKDGGIFAFAF-DYRKFGTSKT------DHKNVWTAY---------------TNGERM      1093
```

```
CA_Cas12a       YDKA-EGKFAFTF-DYLDYNVKSE------CGRTLWTVY---------------TVGERF      1089
                :       * :                        *

CR_Cas12a       RWDKNLNQNKGGYTHYTNITENIQELFTKY-----GIDITKDLLTQISTIDEKQNTSFFR      1177
SD_Cas12a       IKP-KNKQGYRED-KRINLTFEMKKLLNEY-----KVSFDLENNLIPNLTSANLKDTFWK      1235
As_Cas12a       VPV-IENHRFTGRYRDLYPANELIALLEEK-----GIVFRDGSNILPKLLENDDS-HAID      1213
TX_Cas12a       YQ-NRRNQKGHWETEEVNVTEKLKALFASDSKTTTVIDYANDDNLIDVILEQDKA-SFFK      1201
Lb_Cas12a       RIFRNPKKNNVFDWEEVCLTSAYKELFNKY-----GINYQQG-DIRALLCEQSDK-AFYS      1125
PC_Cas12a       KNFRNSQKNGQWDSEEFALTEAFKSLFVRY-----EIDYTA--DLKTAIVDEKQK-DFFV      1162
FB_Cas12a       ETKRQKDQNNKFVSTPINLTEKIEDFLGKN-----QIVYGDGNCIKSQIASKDDK-AFFE      1220
MAD7            KRR-FVNGRFSNESDTIDITKDMEKTLEMT-----DINWRDGHDLRQDIIDY----EIVQ      1160
CT_Cas12a       RYI-KEKKRNEL----FDPSKEIKEALTSS-----GIKYDGGQNILPDILRSNNN-GLIY      1142
CA_Cas12a       TYS-RVNREYVR----KVPTDIIYDALQKA-----GISVEGDL---RDRIAESDG-DTLK      1135
                      .            :      :         :

CR_Cas12a       DFIFYFNLICQIRNTDDSEIAKKNGKDDFILSPVE----PFFDSRK---DNGNKLPENGD      1230
SD_Cas12a       ELFFIFKTTLQLRNSVT------NGKEDVLISPVKNAKGEFFVSGT----HNKTLPQDCD      1285
As_Cas12a       TMVALIRSVLQMRNSNA------ATGEDYINSPVRDLNGVCFDSRF----QNPEWPMDAD      1263
TX_Cas12a       ELLWLLKLTMTLRHSKI------KSEDDFILSPVKNEQGEFYDSRK----AGEVWPKDAD      1251
Lb_Cas12a       SFMALMSLMLQMRNSIT-----GRTDVDFLISPVKNSDGIFYDSRNYEAQENAILPKNAD      1180
PC_Cas12a       DLLKLFKLTVQMRNSWK------EKDLDYLISPVAGADGRFFDTRE----GNKSLPKDAD      1212
FB_Cas12a       TLLYWFKMTLQMRNSET------RTDIDYLISPVMNDNGTFYNSRDYEKLENPTLPKDAD      1274
MAD7            HIFEIFRLTVQMRNSLS---ELEDRDYDRLISPVLNENNIFYDSAK----AGDALPKDAD      1213
CT_Cas12a       TMYSSFIAAIQMR--------VYDGKEDYIISPIKNSKGEFFRTDP----KRRELPIDAD      1190
CA_Cas12a       SIFYAFKYALDMR--------VENREEDYIQSPVKNASGEFFCSKN----AGKSLPQDSD      1183
                 :   :     :*              * : **:       : :           * : *

CR_Cas12a       DNGAYNIARKGIVILNKISQYSEKNENCEKMKWGDLYVSNIDWDNFV----------         1277
SD_Cas12a       ANGAYHIALKGLMILERNNLVREEKD--T---KKIMAISNVDWFEYVQKRRGVL*--         1334
As_Cas12a       ANGAYHIALKGQLLLNH---LKESKD--L---KLQNGISNQDWLAYIQELRN*----         1307
TX_Cas12a       ANGAYHIALKGLWNLQQINQWEK--GK-----TLNLAIKNQDWFSFIQEKPYQE*--         1298
Lb_Cas12a       ANGAYNIARKVLWAIGQFKKAEDEKLD-----KVKIAISNKEWLEYAQTSVKH*---         1228
PC_Cas12a       ANGAYNIALKGLWALRQIRQTSE--GG-----KLKLAISNKEWLQFVQERSYEKD*-         1260
FB_Cas12a       ANGAYHIAKKGLMLLNKIDQADL--TK-----KVDLSISNRDWLQFVQKNK*-----         1318
MAD7            ANGAYCIALKGLYEIKQITENWKEDGKFS---RDKLKISNKDWFDFIQNKRYL*---         1263
CT_Cas12a       ANGAYNIALRGELTMRAIAEKFDPDSEK----MAKLELKHKDWFEFMQTRGD*----         1238
CA_Cas12a       ANGAYNIALKGILQLRMLSEQYDPNAES----IRLPLITNKAWLTFMQSGMKTWKN*         1235
                 **** ** :    :                      :.:  *  :
```

Supplementary Figure 2 The amino acid sequence alignment of Cas12a proteins. The crossover points were labeled in red. We used Clustal Omega (https://www.ebi.ac.uk/Tools/msa/clustalo/) for the sequence alignment.

a

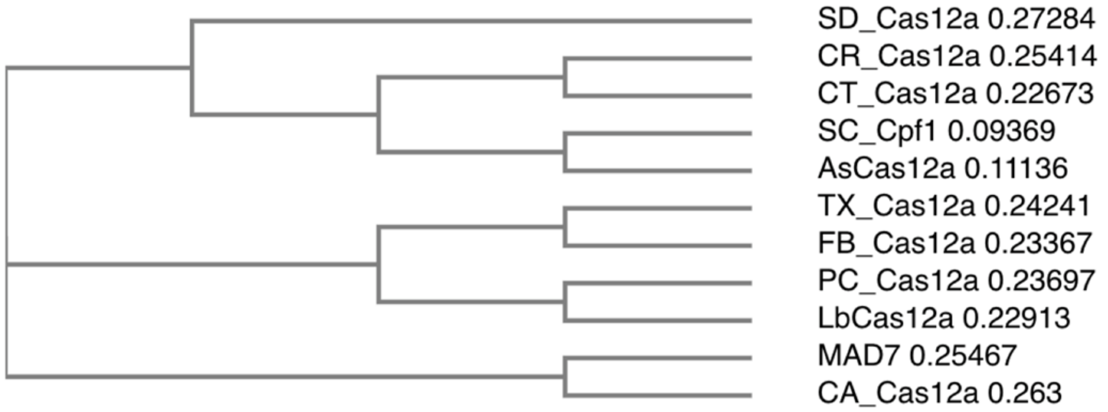

b

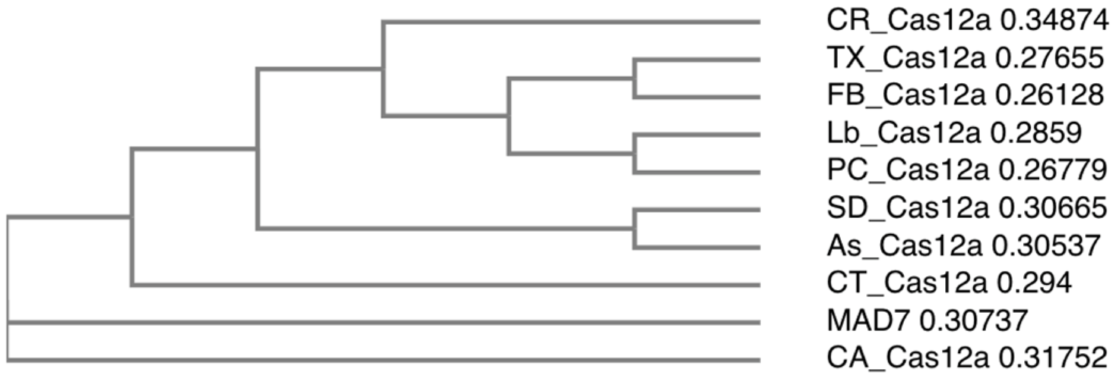

Supplementary Fig. 3 The Phylogenetic Tree for the WT Cas12a and chimera Cas12a like gene (a) and amino acid (b) sequences. We used Clustal Omega for the data analysis and figure generation (https://www.ebi.ac.uk/Tools/msa/clustalo/). As_Cas12a and Lb_Cas12a were reported by Feng Zhang group[21]. M44, M21, M38, M43, and M8 were chimera Cas12a like proteins. The numbers are the distance values which show the number of substitutions (nucleotides or amino acid residues) as a proportion of the length of the alignment (excluding gaps).

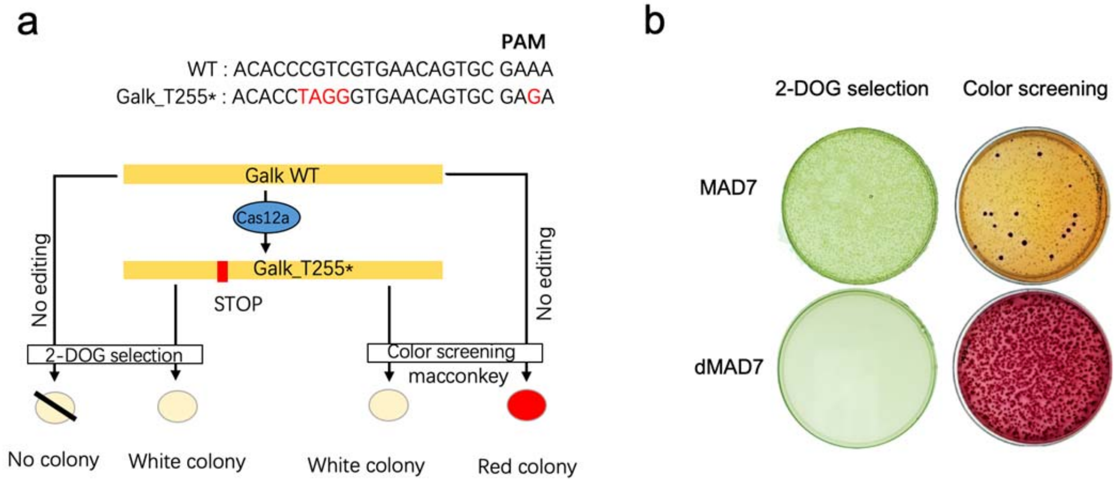

Supplementary Fig. 4 The *galK* based growth selection and colorimetric screen for Cas12a-type chimera library. (a) Schematic showing expected results using 2-DOG selection and Macconkey color screening. Both methods were used to find the functional MAD library variants. (b) Example plates showing the results of 2-DOG selection and Macconkey color screening test using WT MAD7 (top) or dMAD7 (bottom).

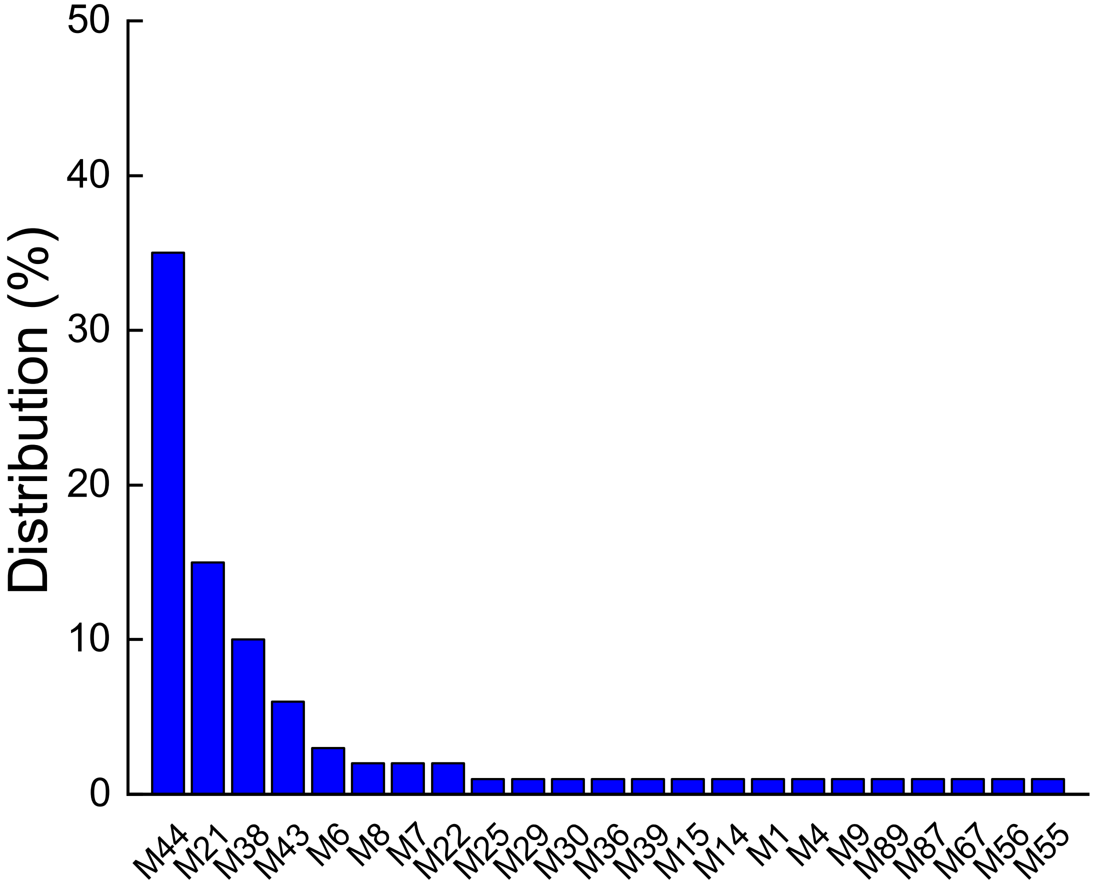

Supplementary Fig. 5 The distribution of functional chimera Cas12a like protein identified from the 2-DOG selection.

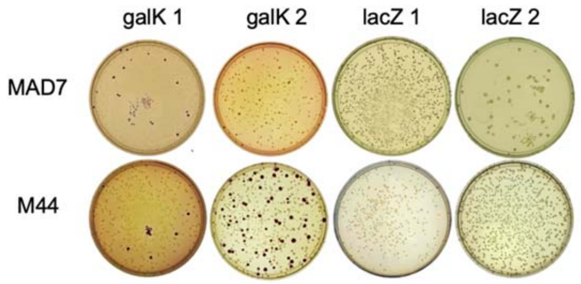

Supplementary Fig. 6 The color screening of MAD7 and M44 with different gRNAs. The edited cells in the galK/lacZ color screening should be shown as white color. The unedited cells in the galK/lacZ color screening should be shown as red color.

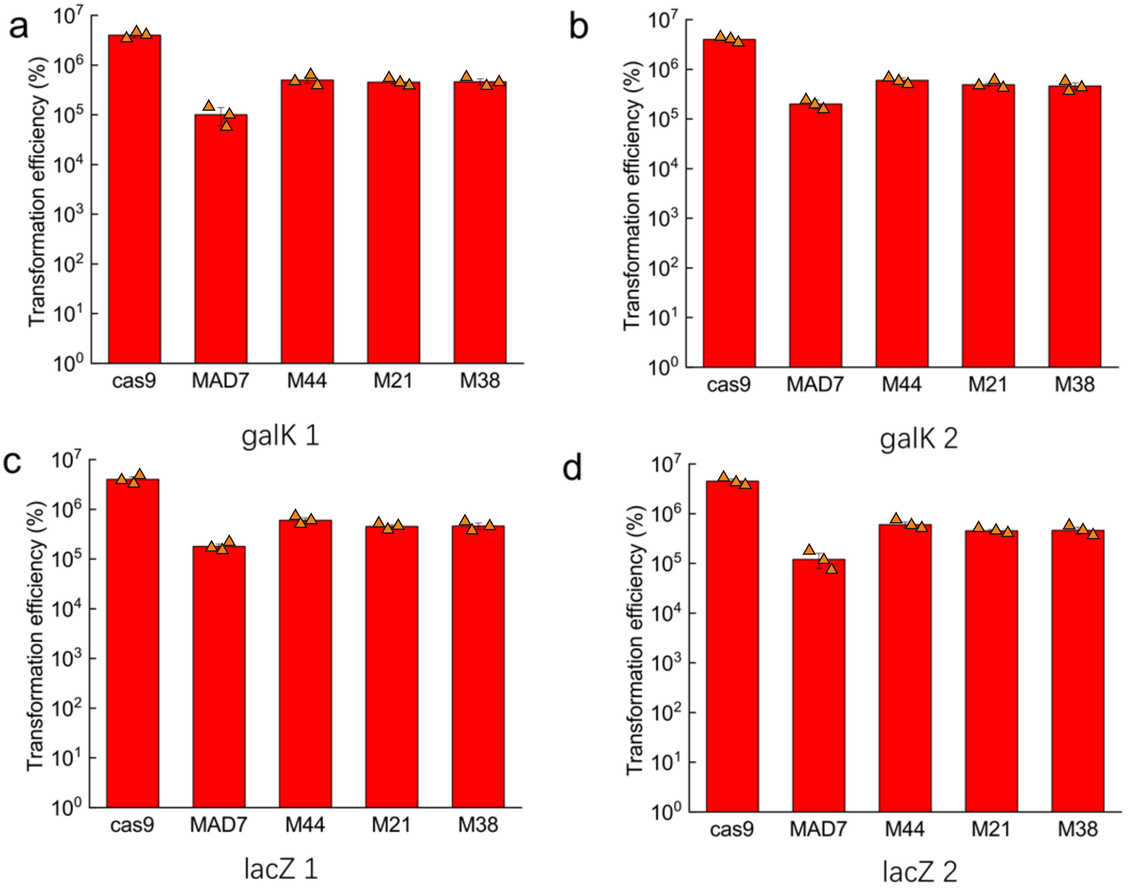

Supplementary Fig. 7 The transformation efficiency of different chimera variants using different gRNA. The gRNA used in the test were (a) galK1 (b) galK2 (c) lacZ1 (d) lacZ2. Transformation efficiency is defined as the number of colony forming units (cfu) per µg of gRNA plasmid.

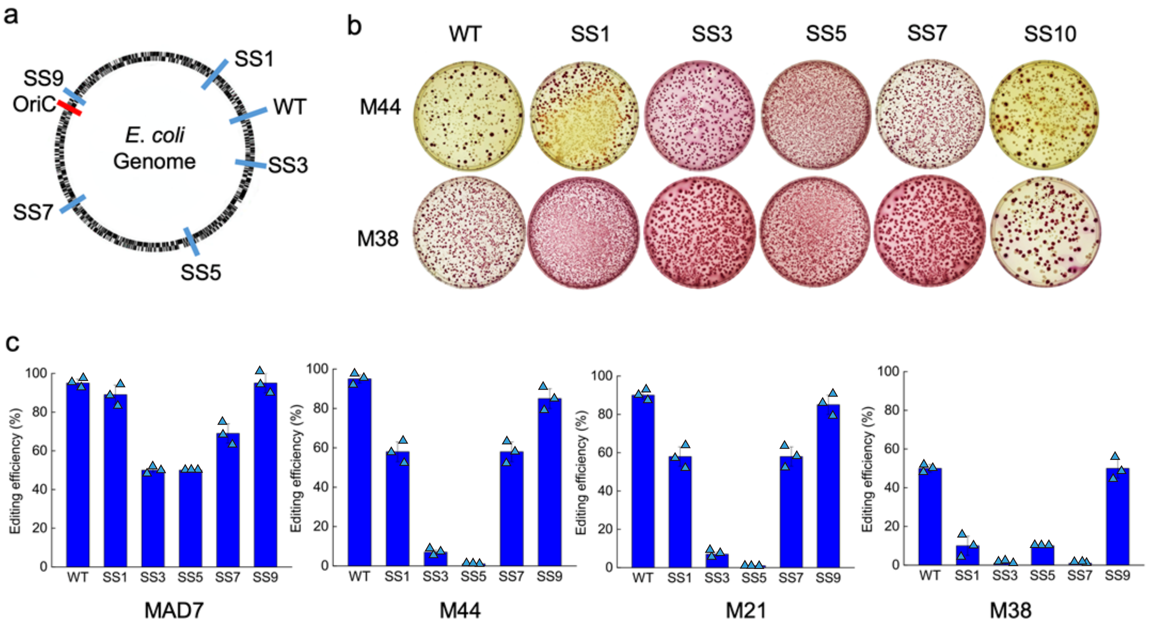

Supplementary Fig. 8 The genome editing test in the different genomic positions for chimera library variants. (a) The targeted genomic position. We integrated *galK* gene individually in the different genomic position (SS1, SS3, SS5, SS7, and SS9) of MG1655△galK. (b) Representative plates for colorimetric screening of GalK activity with variants M44 and M38 in different genomic position. (c) The editing efficiency of chimera library variants in different genomic position.

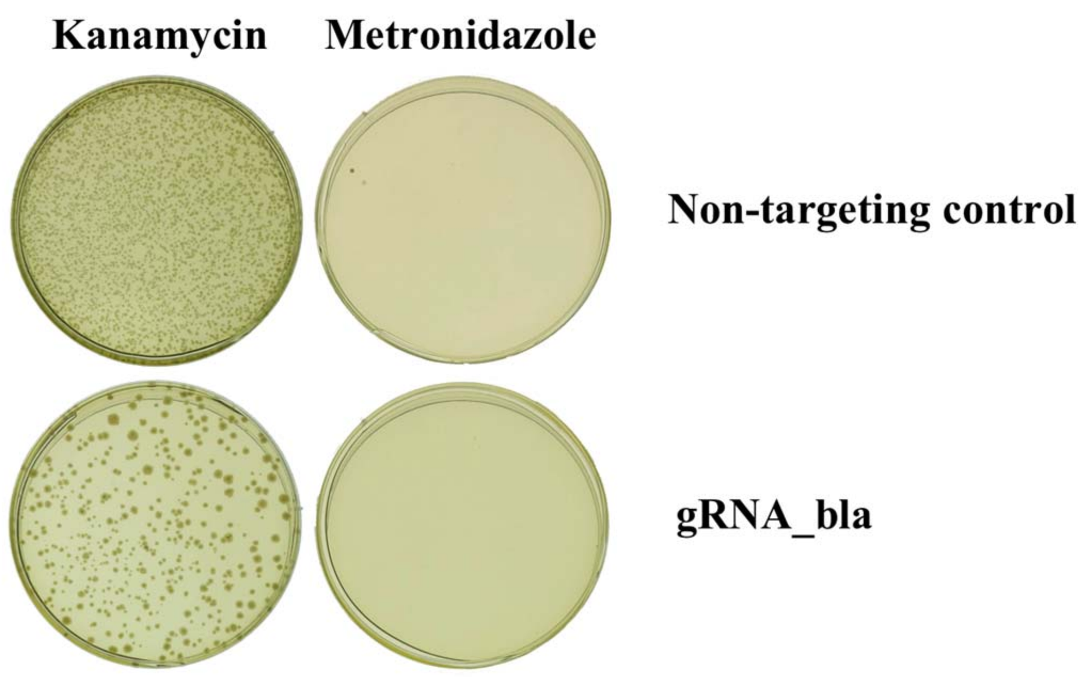

Supplementary Fig. 9 The example for this test using dM44 for DNA binding assay. The behavior of chimera dCas12a variants could be 1) Cutting: cells cannot grow with kanamycin, but cells can grow with metronidazole; 2) DNA binding: cells cannot grow with kanamycin or metronidazole; 3) No DNA binding: cells cannot grow with metronidazole, but cells can grow with kanamycin

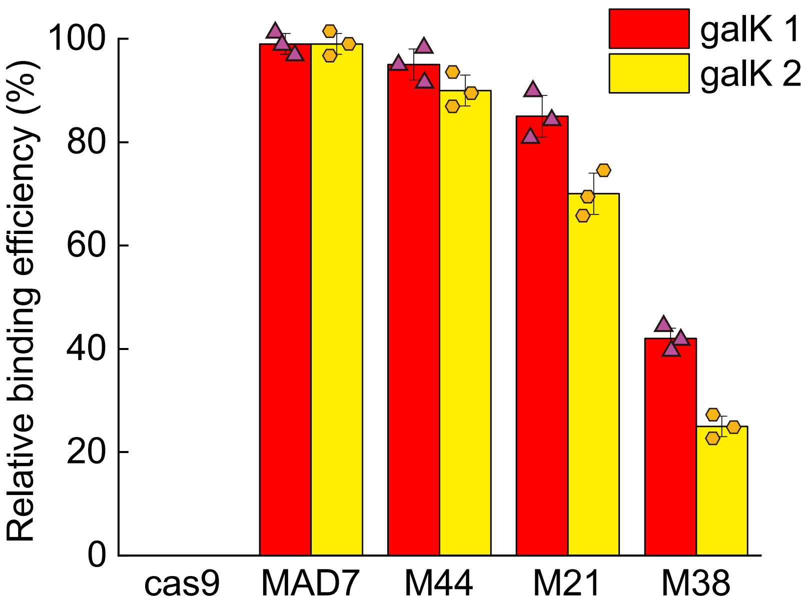

Supplementary Fig. 10 The binding efficiency of dCas12a using different gRNA (galK_1 and galK_2). The binding efficiency was calculated by the following formula.

$$\text{DNA binding efficiency } = \left(1 - \frac{Cells\ \ in\ the\ LB\ agar\ plate\ with\ kanamysin}{Cells\ in\ the\ LB\ agar\ plate\ without\ kanamysin}\right) \times\ 100\%$$

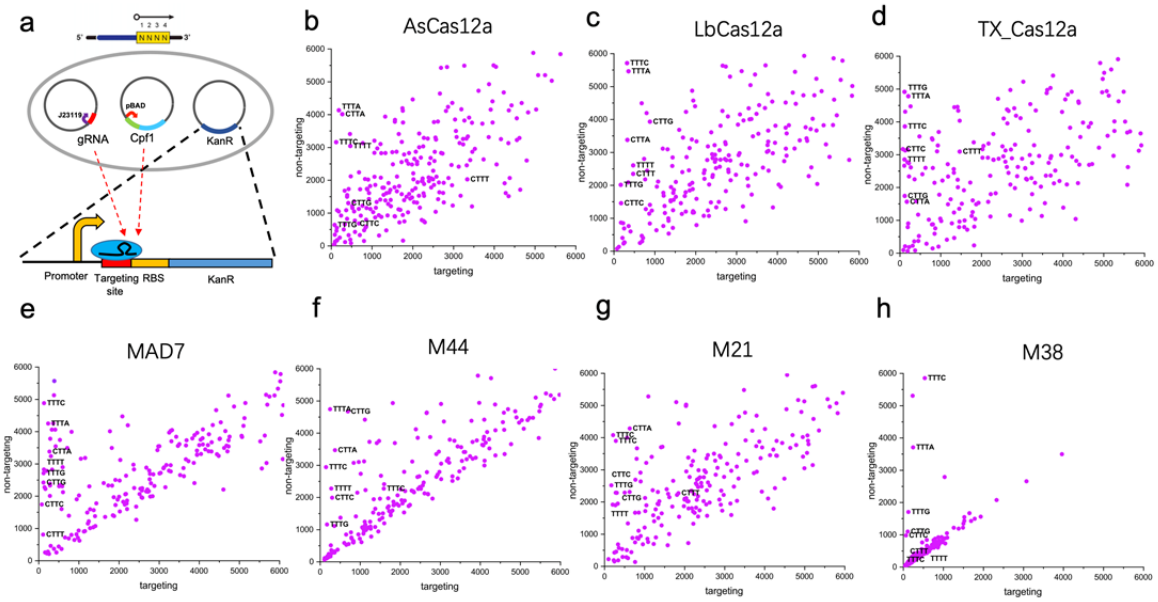

Supplementary Fig. 11 The PAM scan of chimera Cas12a like proteins. (a) The experiment design of PAM scan. We constructed the reporter plasmid containing *KanR* gene encoding for kanamycin resistance and the functional protospacer with NNNN PAM library. We then transformed the chimera Cas12a like proteins and one of two gRNA plasmids individually into the *E. coli* MG1655. One gRNA design is targeted on the *KanR* gene, and another gRNA plasmid is non-targeting control. These two gRNA plasmids were equivalent amount for the transformation. We collected the cells grown on kanamycin media using different gRNA plasmids, and amplified the region of the PAM library from the reported plasmid for the high throughput sequencing. The enrichment score of PAM and accompanying sequence logo for one of two library replicates revealed the PAM specificity among different chimera Cas12a like proteins. (b-h) The first round PAM scan test of different variants. (b) AsCas12a (c) LbCas12a (d) TX_Cas12a (e) MAD7 (f) M44 (g) M21 (h) M38. The X- and Y- axis are normalized reads frequency.

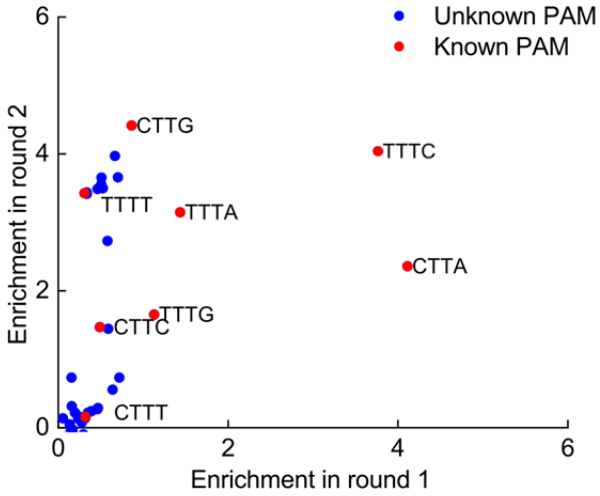

Supplementary Fig. 12 The enrichment score of all 2 rounds of PAM scan for M38. The enrichment score means the frequency change (log_2) of each PAM using different gRNA plasmids (on-targeting and non-targeting gRNAs).

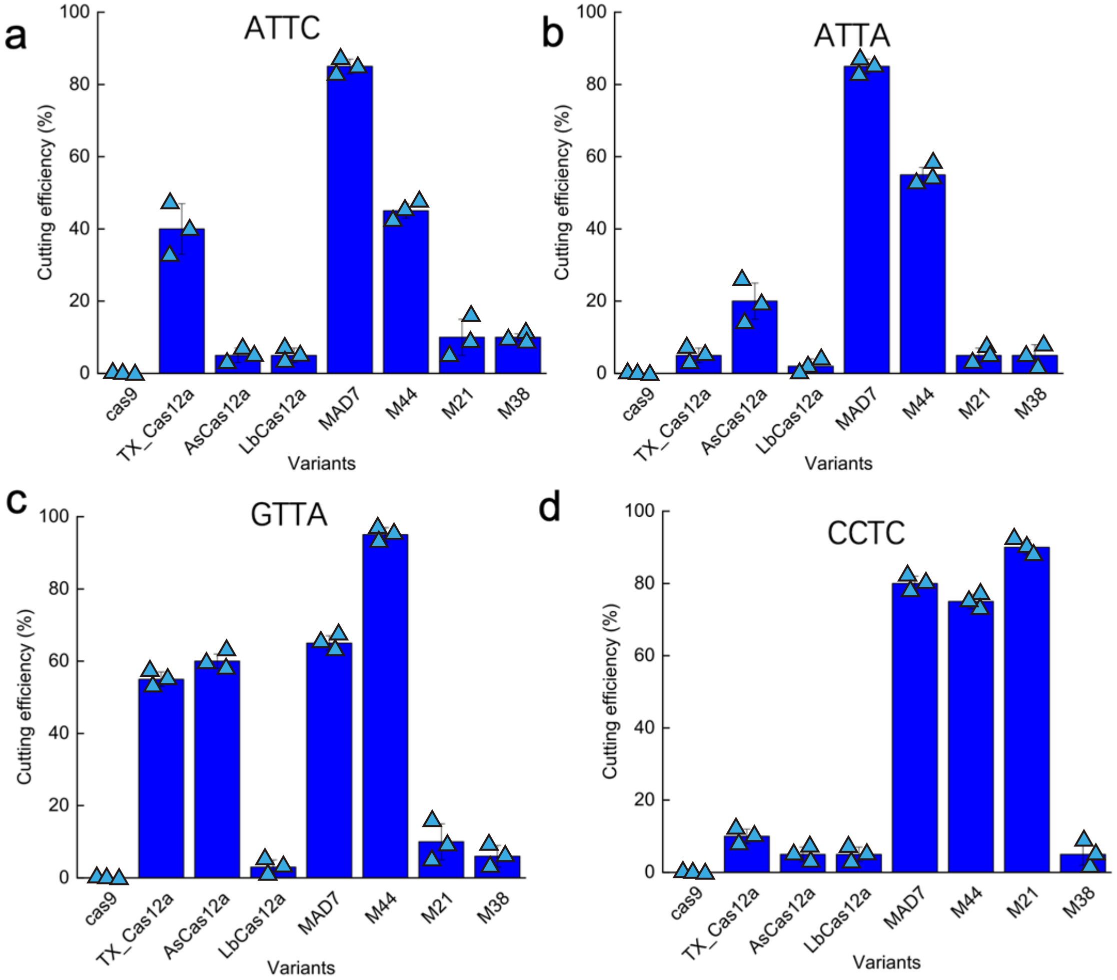

Supplementary Fig. 13 The individual verification of unknown PAMs using different CRISPR associated proteins. (a)ATTC (b) ATTA (c) GTTA (d) CCTC.

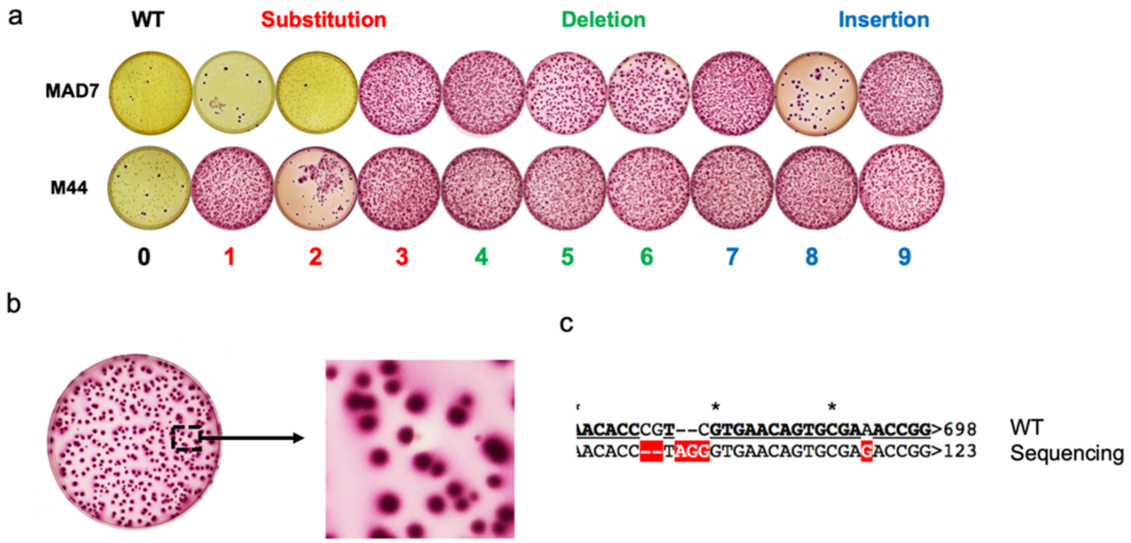

Supplementary Fig. 14 The off-targeting test of MAD7 using 9 plasmids with off-targeting design. (a) Representative plates for colorimetric screening of GalK activity with 9 plasmids with the off-target spacers design. WT means the plasmid with a wild-type spacer design. (b) The color screening of off-target test using design 5. (c) The sequencing verification of white colony.

Supplementary Table 2 The Cas12a proteins sequence homology analysis

Cas12a proteins	Percent Identify (%)
LbCas12a	100.00
AsCas12a	33.80
SD_Cas12a	33.82
CT_Cas12a	34.78
TX_Cas12a	38.30
CA_Cas12a	34.09
PC_Cas12a	42.99
FB_Cas12a	40.44
CR_Cas12a	36.82
SC_Cas12a	33.80
MAD7	35.71

Note: The data analysis was used the BLAST (https://blast.ncbi.nlm.nih.gov/Blast.cgi) from the NCBI database.

We used LbCas12a protein sequences as query sequence.
