# Supplementary material for: Synthetic chimeric nucleases function for efficient genome editing
Source: Nat Commun. 2019 Dec 4;10:5524. doi: 10.1038/s41467-019-13500-y (PMC6892893; doi:10.1038/s41467-019-13500-y)
Supplement: Supplementary file 2 — Supplementary Data 1 [file 41467_2019_13500_MOESM2_ESM.pdf]

Supplementary Table 1 The oligos and sequences used in this study

SD_Cpf1_1	AAGCATTGGCCGTAAGTGCGATTCCGGAAAGGAGATATACatgtcatcgctcacgaaattcactaacaaatactctaaacagctcaccattaagaatgaactcatcccagttggcaaaacactggagaacatcaaagagaatggtctgatagatggcgacgaacagctgaatgagaattatcagaaggcgaaaattattgtggatgattttctgcgggacttcattaataaagcactgaataatacgcagatcgggaactggcgcgaactggcggatgcccttaataaagaggatgaagataacatcgagaaattgcaggataaaattcggggaatcattgtatccaaatttgaaacgtttgatctgtttagcagctattctattaagaaagatgaaaagattattgacgacgacaatgatgttgaagaagaggaactggatctgggcaagaagaccagctcatttaaatacatatttaaaaaaaacctgtttaagttagtgttgccatcctacctgaaaaccacaaaccaggacaagctgaagattattagctcgtttgataatttttcaacgtacttccgcgggttctttgaaaaccggaaaaacatttttaccaagaaaccgatctccacaagtattgcgtatcgcattgttcatgataacttcccgaaattccttgataacattcgttgttttaatgtgtggcagacggaatgcccgcaactaatcgtgaaagcagataactatctgaaaagcaaaaatgttatagcgaaagataaaagtttggcaaactattttaccgtgggcgcgtatgactatttcctgtctcagaatggtatagattttacaacaatattataggtggactgccagcgttcgccggccatgagaaaatccaaggtctcaatgaattcatcaatcaagagtgccaaaaagacagcgagctgaaaagtaagctgaaaaaccgtcacgcgttcaaaatggcggtaCTTCACAAACAGATTCTATGCATTGCGGACACTAGCTATGAG
CT_Cpf1_1	AAGCATTGGCCGTAAGTGCGATTCCGGAAAGGAGATATACatgaacaactacgacgaattcaccaaactgtacccgatccagaaaaccatccgtttcgaactgaaaccgcagggtcgtaccatggaacacctggaaaccttcaacttcttcgaagaagaccgtgaccgtgcggaaaaatacaaaatcctgaaagaagcgatcgacgaataccacaaaaaattcatcgacgaacacctgaccaacatgtctctggactggaactctctgaaacagatctctgaaaaatactacaaatctcgtgaagaaaaagacaaaaaagttttcctgtctgaacagaaacgtatgcgtcaggaaatcgtttctgaattcaaaaaagacgaccgtttcaaagacctgttctctaaaaaactgttctctgaactgctgaaagaagaaatctacaaaaaaggtaaccaccaggaaatcgacgcgctgaaatctttcgacaaattctctggttacttcatcggtctgcacgaaaaccgtaaaaacatgtactctgacggtgacgaaatcaccgcgatctctaaccgtatcgttaacgaaaacttcccgaaattcctggacaacctgcagaaataccaggaagcgcgtaaaaaatacccggaatggatcatcaaagcggaatctgcgctggttgcgcacaacatcaaaatggacgaagttttctctctggaatacttcaacaaagttctgaaccaggaaggtatccagcgttacaacctggcgctgggtggttacgttaccaaatctggtgaaaaaatgatgggtctgaacgacgcgctgaacctggcgcaccagtctgaaaaatcttctaaaggtcgtatccacatgaccccgCTTCACAAACAGATTCTATGCATTGCGGACACTAGCTATGAG
TX_Cpf1_1	AAGCATTGGCCGTAAGTGCGATTCCGGAAAGGAGATATACatgactaaaacatttgattcagagttttttaatttgtactcgctgcaaaaaacggtacgctttgagttaaaacccgtgggagaaaccgcgtcatttgtggaagactttaaaaacgagggcttgaaacgtgttgtgagcgaagatgaaaggcgagccgtcgattaccagaaagttaaggaaataattgacgattaccatcgggatttcattgaagaaagtttaaattattttccggaacaggtgagtaaagatgctcttgagcaggcgtttcatctttatcagaaactgaaggcagcaaaagttgaggaaagggaaaaagcgctgaaagaatgggaagcgctgcagaaaaagctacgtgaaaaagtggtgaaatgcttctcggactcgaataaagcccgcttctcaaggattgataaaaaggaactgattaaggaagacctgataaattggttggtcgcccagaatcgcgaggatgatatccctacggtcgaaacgtttaacaacttcaccacatattttaccggcttccatgagaatcgtaaaaatatttactccaaagatgatcacgccaccgctattagctttcgccttattcatgaaaatcttccaaagtttttttgacaacgtgattagcttcaataagttgaaagagggtttccctgaattaaaatttgataaagtgaaagaggatttagaagtagattatgatctgaagcatgcgtttgaaatagaatatttcgttaacttcgtgacccaagcgggcatagatcagtataattatctgttaggagggaaaaccctggaggacgggacgaaaaaacaagggatgaatgagcaaattaatctgttcaaacaacagcaaacgcgagataaagcgcgtcagattcccaaactgatccccCTTCACAAACAGATTCTATGCATTGCGGACACTAGCTATGAG

CA_Cpf1_1	AAGCATTGGCCGTAAGTGCGATTCCGGAAAGGAGATATACatgcatacaggcggtcttcttagta<br>tggacgcgaaagagttcacaggtcagtatccgttgtcgaaaacattacgattcgaacttcggccc<br>atcggccgcacgtgggataacctggaggcctcaggctacttagcggaagaccgccatcgtgccga<br>atgttatcctcgtgcgaaagagttattggatgacaaccatcgtgccttcctgaatcgtgtgttgc<br>cacaaatcgatatggattggcacccgattgcggaggccttttgtaaggtacataaaaaccctggt<br>aataaagaacttgcccaggattacaaccttcagttgtcaaagcgccgtaaggagatcagcgcata<br>tcttcaggatgcagatggctataaaggcctgttcgcgaagcccgccttagacgaagctatgaaaa<br>ttgcgaaagaaaacgggaacgaaagtgatattgaggttctcgaagcgtttaacggttttagcgta<br>tacttcaccggttatcatgagtcacgcgagaacatttatagcgatgaggatatggtgagcgtagc<br>ctaccgaattactgaggataatttcccgcgctttgtctcaaacgctttgatctttgataaattaa<br>acgaaagccatccggatattatctctgaagtatcgggcaatcttggagttgatgacattggtaag<br>tactttgacgtgtcgaactataacaattttctttcccaggccggtatagatgactacaatcacat<br>tattggcggccatacaaccgaagacggactgatacaagcgtttaatgtcgtattgaacttacgtc<br>accaaaaagaccctggctttgaaaaaattcagttcaaacagCTTCACAAACAGATTCTATGCATT<br>GCGGACACTAGCTATGAG
PC_Cpf1_1	AAGCATTGGCCGTAAGTGCGATTCCGGAAAGGAGATATACatggatagtttgaaagatttcacca<br>atctgtaccctgtcagtaagacattgagatttgaattaaagcccgttggaaagactttagaaaat<br>atcgagaaagcaggtattttgaaagaggatgagcatcgtgcagaaagttatcggagggtgaagaa<br>aataattgatacttatcataaggtatttatcgattcttctcttgaaaatatggctaaaatgggta<br>ttgagaatgaaataaaagcaatgctccaaagtttctgcgaattgtataaaaaagatcatcgcact<br>gagggtgaagacaaggcattagataaaattcgagcagtacttcgtggcctgattgttggggcttt<br>cactggtgtttgcggaagacgggaaaatacagtccaaaacgagaagtacgagagtttgttcaaag<br>aaaagttgataaaagaaattttacctgattttgtgctctctactgaggctgaaagcttgcctttc<br>tctgttgaagaagctacgaggtcactgaaggagtttgatagctttacatcctactttgctggttt<br>ttacgagaatagaaagaatatatactcgacgaaacctcaatccactgccattgcttatcgtctta<br>ttcatgagaacttgccgaagttcattgataatattcttgtttttcagaagatcaaagagcctata<br>gccaaagagctggaacatattcgtgcggacttttctgccggggggtacataaaaaaggatgagag<br>attggaggatattttttcgttgaactattatatccacgtgttatctcaggctgggatcgaaaaat<br>ataacgcattgattgggaagattgtgacagaaggagatggagagatgaaagggctcaatgaacac<br>atcaacctttacaaccaacaaagaggcagagaggatcggctccctctttttaggcctCTTCACAA<br>ACAGATTCTATGCATTGCGGACACTAGCTATGAG
FB_Cpf1_1	AAGCATTGGCCGTAAGTGCGATTCCGGAAAGGAGATATACatgaccaataaattcactaaccagt<br>attctctctctaagaccctgcgctttgaactgattccgcaggggaaaaccttggagttcattcaa<br>gaaaaaggcctcttgtctcaggataaacagagggctgaatcttaccaagaaatgaagaaaactat<br>tgataagtttcataaatatttcattgatttagccttgtctaacgccaaattaactcacttggaaa<br>cgtatctggagttatacaacaaatctgccgaaactaagaaagaacagaaatttaaagacgatttg<br>aaaaaagtacaggacaatctgcgtaaagaaattgtcaaatccttcagtgacggcgatgctaaaag<br>catttttgccattctggacaaaaaagagttgattactgtggaattagaaaagtggtttgaaaaca<br>atgagcagaaagacatctacttcgatgagaaattcaaaactttcaccacctattttacaggattt<br>catcaaaaccggaagaacatgtactcagtagaaccgaactccacggccattgcgtatcgtttgat<br>ccatgagaatctgcctaaatttctggagaatgcgaaagcctttgaaaagattaagcaggtcgaat<br>cgctgcaagtgaattttcgtgaactcatgggcgaatttggtgacgaaggtctaatcttcgttaac<br>gaactggaagaaatgtttcagattaattactacaatgacgtgctatcgcagaacggtatcacaat<br>ctacaatagtattatctcagggttcacaaaaaacgatataaaatacaaaggcctgaacgagtata<br>tcaataactacaaccaaacaaaggacaaaaaggataggcttccgaaactgaagcagCTTCACAAA<br>CAGATTCTATGCATTGCGGACACTAGCTATGAG

CR_Cpf1_1	AAGCATTGGCCGTAAGTGCGATTCCGGAAAGGAGATATACatgtctttcgactctttcaccaacc<br>tgtactctctgtctaaaaccctgaaattcgaaatgcgtccggttggtaacacccagaaaatgctg<br>gacaacgcgggtgttttcgaaaaagacaaactgatccagaaaaaatacggtaaaaccaaaccgta<br>cttcgaccgtctgcaccgtgaattcatcgaagaagcgctgaccggtgttgaactgatcggtctgg<br>acgaaaacttccgtaccctggttgactggcagaaagacaaaaaaaacaacgttgcgatgaaagcg<br>tacgaaaactctctgcagcgtctgcgtaccgaaatcggtaaaatcttcaacctgaaagcggaaga<br>ctgggttaaaaacaaatacccgatcctgggtctgaaaaacaaaaacaccgacatcctgttcgaag<br>aagcggttttcggtatcctgaaagcgcgttacggtgaagaaaaagacaccttcatcgaagttgaa<br>gaaatcgacaaaaccggtaaatctaaaatcaaccagatctctatcttcgactcttggaaaggttt<br>caccggttacttcaaaaaattcttcgaaacccgtaaaaacttctacaaaaacgacggtacctcta<br>ccgcgatcgcgacccgtatcatcgaccagaacctgaaacgtttcatcgacaacctgtctatcgtt<br>gaatctgttcgtcagaaagttgacctggcggaaaccgaaaaatctttctctatctctctgtctca<br>gttcttctctatcgacttctacaacaaatgcctgctgcaggacggtatcgactactacaacaaaa<br>tcatcggtggtgaaaccctgaaaaacggtgaaaaactgatcggtctgaacgaactgatcaaccag<br>taccgtcagaacaacaaagaccagaaaatcccgttcttcaaactgCTTCACAAACAGATTCTATG<br>CATTGCGGACACTAGCTATGAG
SC_Cpf1_1	AAGCATTGGCCGTAAGTGCGATTCCGGAAAGGAGATATACatgacccagttcgaaggtttcacca<br>acctgtaccaggtttctaaaaccctgcgtttcgaactgatcccgcagggtaaaaccctgaaacac<br>atccaggaacagggtttcatcgaagaagacaaagcgcgtaacgaccactacaaagaactgaaacc<br>gatcatcgaccgtatctacaaaacctacgcggaccagtgcctgcagctggttcagctggactggg<br>aaaacctgtctgcggcgatcgactcttaccgtaaagaaaaaaccgaagaaacccgtaacgcgctg<br>atcgaagaacaggcgacctaccgtaacgcgatccacgactacttcatcggtcgtaccgacaacct<br>gaccgacgcgatcaacaaacgtcacgcggaaatctacaaaggtctgttcaaagcggaactgttca<br>acggtaaagttctgaaacagctgggtaccgttaccaccaccgaacacgaaaacgcgctgctgcgt<br>tctttcgacaaattcaccacctacttctctggtttctacgaaaaccgtaaaaacgttttctctgc<br>ggaagacatctctaccgcgatcccgcaccgtatcgttcaggacaacttcccgaaattcaaagaaa<br>actgccacatcttcacccgtctgatcaccgcggttccgtctctgcgtgaacacttcgaaaacgtt<br>aaaaaagcgatcggtatcttcgtttctacctctatcgaagaagttttctctttcccgttctacaa<br>ccagctgctgacccagacccagatcgacctgtacaaccagctgctgggtggtatctctcgtgaag<br>cgggtaccgaaaaaatcaaaggtctgaacgaagttctgaacctggcgatccagaaaaacgacgaa<br>accgcgcacatcatcgcgtctctgccgcaccgtttcatcccgCTTCACAAACAGATTCTATGCAT<br>TGCGGACACTAGCTATGAG

SD_Cpf1_2	AAGCATTGGCCGTAAGTGCGATTCCGGAAAGGAGATATACatgtcatcgctcacgaaattcacta<br>acaaatactctaaacagctcaccattaagaatgaactcatcccagttggcaaaacactggagaac<br>atcaaagagaatggtctgatagatggcgacgaacagctgaatgagaattatcagaaggcgaaaat<br>tattgtggatgattttctgcgggacttcattaataaagcactgaataatacgcagatcgggaact<br>ggcgcgaactggcggatgcccttaataaagaggatgaagataacatcgagaaattgcaggataaa<br>attcggggaatcattgtatccaaatttgaaacgtttgatctgtttagcagctattctattaagaa<br>agatgaaaagattattgacgacgacaatgatgttgaagaagaggaactggatctgggcaagaaga<br>ccagctcatttaaatacatatttaaaaaaaacctgtttaagttagtgttgccatcctacctgaaa<br>accacaaaccaggacaagctgaagattattagctcgtttgataatttttcaacgtacttccgcgg<br>gttctttgaaaaccggaaaaacatttttaccaagaaaccgatctccacaagtattgcgtatcgca<br>ttgttcatgataacttcccgaaattccttgataacattcgttgttttaatgtgtggcagacggaa<br>tgcccgcaactaatcgtgaaagcagataactatctgaaaagcaaaaatgttatagcgaaagataa<br>aagtttggcaaactattttaccgtgggcgcgtatgactatttcctgtctcagaatggtatagatt<br>tttacaacaatattataggtggactgccagcgttcgccggccatgagaaaatccaaggtctcaat<br>gaattcatcaatcaagagtgccaaaaagacagcgagctgaaaagtaagctgaaaaaccgtcacgc<br>gttcaaaatggcggtactgttcaaacagatactcagcgatcgtgaaaaaagttttgtaattgatg<br>agttcgagtcggatgctcaagttattgacgccgttaaaaacttttacgccgaacagtgcaaagat<br>aacaatgttatttttaacttattaaatcttatcaagaatatcgctttcttaagtgatgacgaact<br>ggacggcatattcattgaagggaaatacctgtcgagcgttagtcaaaaactctatagcgattggt<br>caaaattacgtaacgacattgaggattcggctaactctaaacaaggcaataaagagctggccaag<br>aagatcaaaaccaacaaaggggatgtagaaaaagcgatctcgaaatatgagttctcgctgtcgga<br>actgaactcgattgtacatgataacaccaagttttctgacctccttagttgtacactgcataagg<br>tggcttctgagaaactggtgaaggtcaatgaaggcgactggccgaaacatctcaagaataatgaa<br>gagaaacaaaaaatcaaagagccgcttgatgctctgctggagatctataatacacttctgatttt<br>taactgcaaaagcttcaataaaaacggcaacttctatgtcgactatgatcgttgcatcaatgaac<br>tgagttcggtcgtgtatctgtataataaaacacgtaactattgcactaaaAAACCGTACAGCACG<br>AAAAAGATTAAATTGAACTTTGG

CT_Cpf1_2	AAGCATTGGCCGTAAGTGCGATTCCGGAAAGGAGATATACatgaacaactacgacgaattcacca<br>aactgtacccgatccagaaaaccatccgtttcgaactgaaaccgcagggtcgtaccatggaacac<br>ctggaaaccttcaacttcttcgaagaagaccgtgaccgtgcggaaaaatacaaaatcctgaaaga<br>agcgatcgacgaataccacaaaaaattcatcgacgaacacctgaccaacatgtctctggactgga<br>actctctgaaacagatctctgaaaaatactacaaatctcgtgaagaaaaagacaaaaaagttttc<br>ctgtctgaacagaaacgtatgcgtcaggaaatcgtttctgaattcaaaaaagacgaccgtttcaa<br>agacctgttctctaaaaaactgttctctgaactgctgaaagaagaaatctacaaaaaaggtaacc<br>accaggaaatcgacgcgctgaaatctttcgacaaattctctggttacttcatcggtctgcacgaa<br>aaccgtaaaaacatgtactctgacggtgacgaaatcaccgcgatctctaaccgtatcgttaacga<br>aaacttcccgaaattcctggacaacctgcagaaataccaggaagcgcgtaaaaaatacccggaat<br>ggatcatcaaagcggaatctgcgctggttgcgcacaacatcaaaatggacgaagttttctctctg<br>gaatacttcaacaaagttctgaaccaggaaggtatccagcgttacaacctggcgctgggtggtta<br>cgttaccaaatctggtgaaaaaatgatgggtctgaacgacgcgctgaacctggcgcaccagtctg<br>aaaaatcttctaaaggtcgtatccacatgaccccgctgttcaaacagatcctgtctgaaaaagaa<br>tctttctcttacatcccggacgttttcaccgaagactctcagctgctgccgtctatcggtggttt<br>cttcgcgcagatcgaaaacgacaaagacggtaacatcttcgaccgtgcgctggaactgatctctt<br>cttacgcggaatacgacaccgaacgtatctacatccgtcaggcggacatcaaccgtgtttctaac<br>gttatcttcggtgaatggggtaccctgggtggtctgatgcgtgaatacaaagcggactctatcaa<br>cgacatcaacctggaacgtacctgcaaaaaagttgacaaatggctggactctaaagaattcgcgc<br>tgtctgacgttctggaagcgatcaaacgtaccggtaacaacgacgcgttcaacgaatacatctct<br>aaaatgcgtaccgcgcgtgaaaaaatcgacgcggcgcgtaaagaaatgaaattcatctctgaaaa<br>aatctctggtgacgaagaatctatccacatcatcaaaaccctgctggactctgttcagcagttcc<br>tgcacttcttcaacctgttcaaagcgcgtcaggacatcccgctggacggtgcgttctacgcggaa<br>ttcgacgaagttcactctaaactgttcgcgatcgttccgctgtacaacaaagttcgtaactacct<br>gaccaaaAAACCGTACAGCACGAAAAAGATTAAATTGAACTTTGG

TX_Cpf1_2	AAGCATTGGCCGTAAGTGCGATTCCGGAAAGGAGATATACatgactaaaacatttgattcagagt<br>tttttaatttgtactcgctgcaaaaaacggtacgctttgagttaaaacccgtgggagaaaccgcg<br>tcatttgtggaagactttaaaaacgagggcttgaaacgtgttgtgagcgaagatgaaaggcgagc<br>cgtcgattaccagaaagttaaggaaataattgacgattaccatcgggatttcattgaagaaagtt<br>taaattattttccggaacaggtgagtaaagatgctcttgagcaggcgtttcatctttatcagaaa<br>ctgaaggcagcaaaagttgaggaaagggaaaaagcgctgaaagaatgggaagcgctgcagaaaaa<br>gctacgtgaaaaagtggtgaaatgcttctcggactcgaataaagcccgcttctcaaggattgata<br>aaaaggaactgattaaggaagacctgataaattggttggtcgcccagaatcgcgaggatgatatc<br>cctacggtcgaaacgtttaacaacttcaccacatattttaccggcttccatgagaatcgtaaaaa<br>tatttactccaaagatgatcacgccaccgctattagctttcgccttattcatgaaaatcttccaa<br>agtttttgacaacgtgattagcttcaataagttgaaagagggtttccctgaattaaaatttgat<br>aaagtgaaagaggatttagaagtagattatgatctgaagcatgcgtttgaaatagaatatttcgt<br>taacttcgtgacccaagcgggcatagatcagtataattatctgttaggagggaaaaccctggagg<br>acgggacgaaaaaacaagggatgaatgagcaaattaatctgttcaaacaacagcaaacgcgagat<br>aaagcgcgtcagattcccaaactgatccccctgttcaaacagattcttagcgaaaggactgaaag<br>ccagtcctttattcctaaacaatttgaaagtgatcaggagttgttcgattcactgcagaagttac<br>ataataactgccaggataaattcaccgtgctgcaacaagccattctcggtctggcagaggcggat<br>cttaagaaggtcttcatcaaaacctctgatttaaatgccttatctaacaccattttcgggaatta<br>cagcgtcttttccgatgcactgaacctgtataaagaaagcctgaaaacgaaaaaagcgcaggagg<br>cttttgagaaactaccggcccattctattcacgacctcattcaatacttggaacagttcaattcc<br>agcctggacgcggaaaaacaacagagcaccgacaccgtcctgaactacttcatcaagaccgatga<br>attatattctcgcttcattaaatccactagcgaggctttcactcaggtgcagcctttgttcgaac<br>tggaagccctgtcatctaagcgccgcccaccggaatcggaagatgaaggggcaaaagggcaggaa<br>ggcttcgagcagatcaagcgtattaaagcttacctggatacgcttatggaagcggtacactttgc<br>aaagccgttgtatcttgttaagggtcgtaaaatgatcgaagggctcgataaagaccagtcctttt<br>atgaagcgtttgaaatggcgtaccaagaacttgaatcgttaatcattcctatctataacaaagcg<br>cggagctatctgtcgcggAAACCGTACAGCACGAAAAAGATTAAATTGAACTTTGG

CA_Cpf1_2	AAGCATTGGCCGTAAGTGCGATTCCGGAAAGGAGATATACatgcatacaggcggtcttcttagta<br>tggacgcgaaagagttcacaggtcagtatccgttgtcgaaaacattacgattcgaacttcggccc<br>atcggccgcacgtgggataacctggaggcctcaggctacttagcggaagaccgccatcgtgccga<br>atgttatcctcgtgcgaaagagttattggatgacaaccatcgtgccttcctgaatcgtgtgttgc<br>cacaaatcgatatggattggcacccgattgcggaggccttttgtaaggtacataaaaaccctggt<br>aataaagaacttgcccaggattacaaccttcagttgtcaaagcgccgtaaggagatcagcgcata<br>tcttcaggatgcagatggctataaaggcctgttcgcgaagcccgccttagacgaagctatgaaaa<br>ttgcgaaagaaaacgggaacgaaagtgatattgaggttctcgaagcgtttaacggttttagcgta<br>tacttcaccggttatcatgagtcacgcgagaacatttatagcgatgaggatatggtgagcgtagc<br>ctaccgaattactgaggataatttcccgcgctttgtctcaaacgctttgatctttgataaattaa<br>acgaaagccatccggatattatctctgaagtatcgggcaatcttggagttgatgacattggtaag<br>tactttgacgtgtcgaactataacaattttctttcccaggccggtatagatgactacaatcacat<br>tattggcggccatacaaccgaagacggactgatacaagcgtttaatgtcgtattgaacttacgtc<br>accaaaaagaccctggctttgaaaaaattcagttcaaacagctctacaaacaaatcctgagcgtg<br>cgtaccagcaaaagctacatcccgaaacagtttgacaactctaaggagatggttgactgcatttg<br>cgattatgtcagcaaaatagagaaatccgaaacagtagaacgggccctgaaactagtccgtaata<br>tcagttctttcgacttgcgcgggatctttgtcaataaaaagaacttgcgcatactgagcaacaaa<br>ctgataggagattgggacgcgatcgaaaccgcattgatgcatagttcttcatcagaaaacgataa<br>gaaaagcgtatatgatagcgcggaggcttttacgttggatgacatcttttcaagcgtgaaaaaat<br>tttctgatgcctctgccgaagatattggcaacagggcggaagacatctgtagagtgataagtgag<br>acggcccctttttatcaacgatctgcgagcggtggacctggatagcctgaacgacgatggttatga<br>agcggccgtctcaaaaattcgggagtcgctggagccttatatggatcttttccatgaactggaaa<br>ttttctcggttggcgatgagttcccaaaatgcgcagcattttacagcgaactggaggaagtcagc<br>gaacagctgatcgaaattattccgttattcaacaaggcgcgttcgttctgcacccggAAACCGTA<br>CAGCACGAAAAAGATTAAATTGAACTTTGG

PC_Cpf1_2	AAGCATTGGCCGTAAGTGCGATTCCGGAAAGGAGATATACatggatagtttgaaagatttcacca<br>atctgtaccctgtcagtaagacattgagatttgaattaaagcccgttggaaagactttagaaaat<br>atcgagaaagcaggtattttgaaagaggatgagcatcgtgcagaaagttatcggagggtgaagaa<br>aataattgatacttatcataaggtatttatcgattcttctcttgaaaatatggctaaaatgggta<br>ttgagaatgaaataaaagcaatgctccaaagtttctgcgaattgtataaaaaagatcatcgcact<br>gagggtgaagacaaggcattagataaaattcgagcagtacttcgtggcctgattgttggggcttt<br>cactggtgtttgcggaagacgggaaaatacagtccaaaacgagaagtacgagagtttgttcaaag<br>aaaagttgataaaagaaattttacctgattttgtgctctctactgaggctgaaagcttgcctttc<br>tctgttgaagaagctacgaggtcactgaaggagtttgatagctttacatcctactttgctggttt<br>ttacgagaatagaaagaatatatactcgacgaaacctcaatccactgccattgcttatcgtctta<br>ttcatgagaacttgccgaagttcattgataatattcttgtttttcagaagatcaaagagcctata<br>gccaaagagctggaacatattcgtgcggacttttctgccgggggtacataaaaaaggatgagag<br>attggaggatatttttttcgttgaactattatatccacgtgttatctcaggctgggatcgaaaaat<br>ataacgcattgattgggaagattgtgacagaaggagatggagagatgaaagggctcaatgaacac<br>atcaacctttacaaccaacaaagaggcagagaggatcggctccctctttttaggcctctttataa<br>acagatattgagtgacagagagcaattatcatacttgcctgagagttttgaaaaagatgaggagc<br>tcctcagggctctaaaagagttctatgatcatatcgcagaagacattctcggacgtactcaacag<br>ttgatgacttctatttcagaatatgatttatctcggatatacgtaaggaacgatagccaattgac<br>tgatatatcaaaaaaaatgttgggagattggaatgctatctacatggctagagaacgagcatatg<br>accacgagcaggctcccaaaagaatcacggcgaaatacgagagggacaggattaaagctcttaaa<br>ggagaagagagtataagtctggcaaatcttaatagttgtattgcctttctggacaatgttagaga<br>ttgccgtgtagatacttatctttccacactgggccagaaggaaggaccacatggtctatctaatc<br>tcgttgagaacgtttttgcctcataccatgaagcagagcaattgttgagctttccataccccgaa<br>gagaataatctgattcaggacaaggacaatgtggtgttaattaagaatcttctcgacaatatcag<br>tgatctgcagaggttcttgaaacctctttggggtatgggagacgaacccgataaagatgaaagat<br>tttatggagagtataattatatccgaggagctctagatcaggtgatccctctgtacaataaggta<br>aggaactacctcactcggAAACCGTACAGCACGAAAAAGATTAAATTGAACTTTGG

FB_Cpf1_2	AAGCATTGGCCGTAAGTGCGATTCCGGAAAGGAGATATACatgaccaataaattcactaaccagt<br>attctctctctaagaccctgcgctttgaactgattccgcaggggaaaaccttggagttcattcaa<br>gaaaaaggcctcttgtctcaggataaacagagggctgaatcttaccaagaaatgaagaaaactat<br>tgataagtttcataaatatttcattgatttagccttgtctaacgccaaattaactcacttggaaa<br>cgtatctggagttatacaacaaatctgccgaaactaagaaagaacagaaatttaaagacgatttg<br>aaaaaagtacaggacaatctgcgtaaagaaattgtcaaatccttcagtgacggcgatgctaaaag<br>catttttgccattctggacaaaaaagagttgattactgtggaattagaaaagtggtttgaaaaca<br>atgagcagaaagacatctacttcgatgagaaattcaaaactttcaccacctattttacaggattt<br>catcaaaaccggaagaacatgtactcagtagaaccgaactccacggccattgcgtatcgtttgat<br>ccatgagaatctgcctaaatttctggagaatgcgaaagcctttgaaaagattaagcaggtcgaat<br>cgctgcaagtgaattttcgtgaactcatgggcgaatttggtgacgaaggtctaatcttcgttaac<br>gaactggaagaaatgtttcagattaattactacaatgacgtgctatcgcagaacggtatcacaat<br>ctacaatagtattatctcagggttcacaaaaaacgatataaaatacaaaggcctgaacgagtata<br>tcaataactacaaccaaacaaaggacaaaaaggataggcttccgaaactgaagcagttatacaaa<br>cagattttatctgacagaatctccctgagctttctgccggatgctttcactgatgggaagcaggt<br>tctgaaagcgattttcgatttttataagattaacttactgagctacacgattgaaggtcaagaag<br>aatctcaaaacttactgctcttgatccgtcaaaccattgaaaatctatcatcgttcgatacgcag<br>aaaatctacctcaaaaacgatactcacctgactacgatctctcagcaggttttcggggattttag<br>tgtattttcaacagctctgaactactggtatgaaaccaaagtcaatccgaaattcgagacggaat<br>attctaaggccaacgaaaaaaaacgtgagattcttgataaagctaaagccgtatttactaaacag<br>gattacttttctattgctttcctgcaggaagttttatcggagtatatcctgaccctggatcatac<br>atctgatatcgttaaaaaacacagcagcaattgcatcgctgactatttcaaaaaccactttgtcg<br>ccaaaaaagaaaacgaaacagacaagactttcgatttcattgctaacatcaccgcaaaataccag<br>tgtattcagggtatcttggaaaacgccgaccaatacgaagacgaactgaaacaagatcagaagct<br>gatcgataatttaaaattcttcttagatgcaatcctggagctgctgcacttcatcaaaccgcttc<br>atttaaagagcgagtccattaccgaaaaggacaccgccttctatgacgtttttgaaaattattat<br>gaagccctctccttgctgactccgctgtataatatggtacgcaattacgtaacccagAAACCGTA<br>CAGCACGAAAAAGATTAAATTGAACTTTGG

CR_Cpf1_2	AAGCATTGGCCGTAAGTGCGATTCCGGAAAGGAGATATACatgtctttcgactctttcaccaacc<br>tgtactctctgtctaaaaccctgaaattcgaaatgcgtccggttggtaacacccagaaaatgctg<br>gacaacgcgggtgttttcgaaaaagacaaactgatccagaaaaaatacggtaaaaccaaaccgta<br>cttcgaccgtctgcaccgtgaattcatcgaagaagcgctgaccggtgttgaactgatcggtctgg<br>acgaaaacttccgtaccctggttgactggcagaaagacaaaaaaaacaacgttgcgatgaaagcg<br>tacgaaaactctctgcagcgtctgcgtaccgaaatcggtaaaatcttcaacctgaaagcggaaga<br>ctgggttaaaaacaaatacccgatcctgggtctgaaaaacaaaaacaccgacatcctgttcgaag<br>aagcggttttcggtatcctgaaagcgcgttacggtgaagaaaaagacaccttcatcgaagttgaa<br>gaaatcgacaaaaccggtaaatctaaaatcaaccagatctctatcttcgactcttggaaaggttt<br>caccggttacttcaaaaaattcttcgaaacccgtaaaaacttctacaaaaacgacggtacctcta<br>ccgcgatcgcgacccgtatcatcgaccagaacctgaaacgtttcatcgacaacctgtctatcgtt<br>gaatctgttcgtcagaaagttgacctggcggaaaccgaaaaatctttctctatctctctgtctca<br>gttcttctctatcgacttctacaacaaatgcctgctgcaggacggtatcgactactacaacaaaa<br>tcatcggtggtgaaaccctgaaaaacggtgaaaaactgatcggtctgaacgaactgatcaaccag<br>taccgtcagaacaacaaagaccagaaaatcccgttcttcaaactgctggacaaacagatcctgtc<br>tgaaaaaatcctgttcctggacgaaatcaaaaacgacaccgaactgatcgaagcgctgtctcagt<br>tcgcgaaaaccgcggaagaaaaaaccaaaatcgttaaaaaactgttcgcggacttcgttgaaaac<br>aactctaaatacgacctggcgcagatctacatctctcaggaagcgttcaacaccatctctaacaa<br>atggacctctgaaaccgaaaccttcgcgaaatacctgttcgaagcgatgaaatctggtaaactgg<br>cgaaatacgaaaaaaaagacaactcttacaaattcccggacttcatcgcgctgtctcagatgaaa<br>tctgcgctgctgtctatctctctggaaggtcacttctggaaagaaaaatactacaaaatctctaa<br>attccaggaaaaaaccaactgggaacagttcctggcgatcttcctgtacgaattcaactctctgt<br>tctctgacaaaatcaacaccaaagacggtgaaaccaaacaggttggttactacctgttcgcgaaa<br>gacctgcacaacctgatcctgtctgaacagatcgacatcccgaaagactctaaagttaccatcaa<br>agacttcgcggactctgttctgaccatctaccagatggcgaaatacttcgcggttgaaaaaaaac<br>gtgcgtggctggcggaatacgaactggactctttctacacccagccggacaccggttacctgcag<br>ttctacgacaacgcgtacgaagacatcgttcaggtttacaacaaactgcgtaactacctgaccaa<br>aAAACCGTACAGCACGAAAAAGATTAAATTGAACTTTGG

SC_Cpf1_2	AAGCATTGGCCGTAAGTGCGATTCCGGAAAGGAGATATACatgacccagttcgaaggtttcacca<br>acctgtaccaggtttctaaaaccctgcgtttcgaactgatcccgcagggtaaaaccctgaaacac<br>atccaggaacagggtttcatcgaagaagacaaagcgcgtaacgaccactacaaagaactgaaacc<br>gatcatcgaccgtatctacaaaacctacgcggaccagtgcctgcagctggttcagctggactggg<br>aaaacctgtctgcggcgatcgactcttaccgtaaagaaaaaaccgaagaaacccgtaacgcgctg<br>atcgaagaacaggcgacctaccgtaacgcgatccacgactacttcatcggtcgtaccgacaacct<br>gaccgacgcgatcaacaaacgtcacgcggaaatctacaaaggtctgttcaaagcggaactgttca<br>acggtaaagttctgaaacagctgggtaccgttaccaccaccgaacacgaaaacgcgctgctgcgt<br>tctttcgacaaattcaccacctacttctctggtttctacgaaaaccgtaaaaacgttttctctgc<br>ggaagacatctctaccgcgatcccgcaccgtatcgttcaggacaacttcccgaaattcaaagaaa<br>actgccacatcttcacccgtctgatcaccgcggttccgtctctgcgtgaacacttcgaaaacgtt<br>aaaaaagcgatcggtatcttcgtttctacctctatcgaagaagttttctctttcccgttctacaa<br>ccagctgctgacccagacccagatcgacctgtacaaccagctgctgggtggtatctctcgtgaag<br>cgggtaccgaaaaaatcaaaggtctgaacgaagttctgaacctggcgatccagaaaaacgacgaa<br>accgcgcacatcatcgcgtctctgccgcaccgtttcatcccgctgttcaaacagatcctgtctga<br>ccgtaacaccctgtctttcatcctggaagaattcaaatctgacgaagaagttatccagtctttct<br>gcaaatacaaaaccctgctgcgtaacgaaaacgttctggaaaccgcggaagcgctgttcaacgaa<br>ctgaactctatcgacctgacccacatcttcatctctcacaaaaaactggaaaccatctcttctgc<br>gctgtgcgaccactgggacaccctgcgtaacgcgctgtacgaacgtcgtatctctgaactgaccg<br>gtaaaatcaccaaatctgcgaaagaaaaagttcagcgttctctgaaacacgaagacatcaacctg<br>caggaaatcatctctgcggcgggtaaagaactgtctgaagcgttcaaacagaaaacctctgaaat<br>cctgtctcacgcgcacgcggcgctggaccagccgctgccgaccaccctgaaaaaacaggaagaaa<br>aagaaatcctgaaatctcagctggactctctgctgggtctgtaccacctgctggactggttcgcg<br>gttgacgaatctaacgaagttgacccggaattctctgcgcgtctgaccggtatcaaactggaaat<br>ggaaccgtctctgtctttctacaacaaagcgcgtaactacgcgaccaaaAAACCGTACAGCACGA<br>AAAAGATTAAATTGAACTTTGG

SD_Cpf1_3	AAGCATTGGCCGTAAGTGCGATTCCGGAAAGGAGATATACatgtcatcgctcacgaaattcacta<br>acaaatactctaaacagctcaccattaagaatgaactcatcccagttggcaaaacactggagaac<br>atcaaagagaatggtctgatagatggcgacgaacagctgaatgagaattatcagaaggcgaaaat<br>tattgtggatgattttctgcgggacttcattaataaagcactgaataatacgcagatcgggaact<br>ggcgcgaactggcggatgcccttaataaagaggatgaagataacatcgagaaattgcaggataaa<br>attcggggaatcattgtatccaaatttgaaacgtttgatctgtttagcagctattctattaagaa<br>agatgaaaagattattgacgacgacaatgatgttgaagaagaggaactggatctgggcaagaaga<br>ccagctcatttaaatacatatttaaaaaaaacctgtttaagttagtgttgccatcctacctgaaa<br>accacaaaccaggacaagctgaagattattagctcgtttgataatttttcaacgtacttccgcgg<br>gttctttgaaaaccggaaaaacatttttaccaagaaaccgatctccacaagtattgcgtatcgca<br>ttgttcatgataacttcccgaaattccttgataacattcgttgttttaatgtgtggcagacggaa<br>tgcccgcaactaatcgtgaaagcagataactatctgaaaagcaaaaatgttatagcgaaagataa<br>aagtttggcaaactattttaccgtgggcgcgtatgactatttcctgtctcagaatggtatagatt<br>tttacaacaatattataggtggactgccagcgttcgccggccatgagaaaatccaaggtctcaat<br>gaattcatcaatcaagagtgccaaaaagacagcgagctgaaaagtaagctgaaaaaccgtcacgc<br>gttcaaaatggcggtactgttcaaacagatactcagcgatcgtgaaaaaagttttgtaattgatg<br>agttcgagtcggatgctcaagttattgacgccgttaaaaacttttacgccgaacagtgcaaagat<br>aacaatgttatttttaacttattaaatcttatcaagaatatcgctttcttaagtgatgacgaact<br>ggacggcatattcattgaagggaaatacctgtcgagcgttagtcaaaaactctatagcgattggt<br>caaaattacgtaacgacattgaggattcggctaactctaaacaaggcaataaagagctggccaag<br>aagatcaaaaccaacaaaggggatgtagaaaaagcgatctcgaaatatgagttctcgctgtcgga<br>actgaactcgattgtacatgataacaccaagttttctgacctccttagttgtacactgcataagg<br>tggcttctgagaaactggtgaaggtcaatgaaggcgactggccgaaacatctcaagaataatgaa<br>gagaaacaaaaaatcaaagagccgcttgatgctctgctggagatctataatacacttctgatttt<br>taactgcaaaagcttcaataaaaacggcaacttctatgtcgactatgatcgttgcatcaatgaac<br>tgagttcggtcgtgtatctgtataataaaacacgtaactattgcactaaaaaccctataacacg<br>gacaagttcaaactcaattttaacagtccgcagctcggtgaaggcttttccaagtcgaaagaaaa<br>tgactgtctgactcttttgtttaaaaaagacgacaactattatgtaggcattatccgcaaaggtg<br>caaaaatcaattttgatgatacacaagcaatcgccgataacaccgacaattgcatctttaaaatg

CT_Cpf1_3	AAGCATTGGCCGTAAGTGCGATTCCGGAAAGGAGATATACatgaacaactacgacgaattcacca<br>aactgtacccgatccagaaaaccatccgtttcgaactgaaaccgcagggtcgtaccatggaacac<br>ctggaaaccttcaacttcttcgaagaagaccgtgaccgtgcggaaaaatacaaaatcctgaaaga<br>agcgatcgacgaataccacaaaaaattcatcgacgaacacctgaccaacatgtctctggactgga<br>actctctgaaacagatctctgaaaaatactacaaatctcgtgaagaaaaagacaaaaaagttttc<br>ctgtctgaacagaaacgtatgcgtcaggaaatcgtttctgaattcaaaaaagacgaccgtttcaa<br>agacctgttctctaaaaaactgttctctgaactgctgaaagaagaaatctacaaaaaaggtaacc<br>accaggaaatcgacgcgctgaaatctttcgacaaattctctggttacttcatcggtctgcacgaa<br>aaccgtaaaaacatgtactctgacggtgacgaaatcaccgcgatctctaaccgtatcgttaacga<br>aaacttcccgaaattcctggacaacctgcagaaataccaggaagcgcgtaaaaaatacccggaat<br>ggatcatcaaagcggaatctgcgctggttgcgcacaacatcaaaatggacgaagttttctctctg<br>gaatacttcaacaaagttctgaaccaggaaggtatccagcgttacaacctggcgctgggtggtta<br>cgttaccaaatctggtgaaaaaatgatgggtctgaacgacgcgctgaacctggcgcaccagtctg<br>aaaaatcttctaaaggtcgtatccacatgaccccgctgttcaaacagatcctgtctgaaaaagaa<br>tctttctcttacatcccggacgttttcaccgaagactctcagctgctgccgtctatcggtggttt<br>cttcgcgcagatcgaaaacgacaaagacggtaacatcttcgaccgtgcgctggaactgatctctt<br>cttacgcggaatacgacaccgaacgtatctacatccgtcaggcggacatcaaccgtgtttctaac<br>gttatcttcggtgaatggggtaccctgggtggtctgatgcgtgaatacaaagcggactctatcaa<br>cgacatcaacctggaacgtacctgcaaaaaagttgacaaatggctggactctaaagaattcgcgc<br>tgtctgacgttctggaagcgatcaaacgtaccggtaacaacgacgcgttcaacgaatacatctct<br>aaaatgcgtaccgcgcgtgaaaaaatcgacgcggcgcgtaaagaaatgaaattcatctctgaaaa<br>aatctctggtgacgaagaatctatccacatcatcaaaaccctgctggactctgttcagcagttcc<br>tgcacttcttcaacctgttcaaagcgcgtcaggacatcccgctggacggtgcgttctacgcggaa<br>ttcgacgaagttcactctaaactgttcgcgatcgttccgctgtacaacaaagttcgtaactacct<br>gaccaaaaacaacctgaacaccaaaaaaatcaaactgaacttcaaaaacccgaccctggcgaacg<br>gttgggaccagaacaaagtttacgactacgcgtctctgatcttcctgcgtgacggtaactactac<br>ctgggtatcatcaacccgaaacgtaaaaaaaacatcaaattcgaacagggttctggtaacggtcc<br>gttctaccgtaaaatggtttacaaacagatcccgggtccgaacaaaaacctgccgcgtgttttcc<br>tgacctctaccaaaggtaaaaaagaatacaaaccgtctaaagaaatcatcgaaggttacgaagcg

TX_Cpf1_3	AAGCATTGGCCGTAAGTGCGATTCCGGAAAGGAGATATACatgactaaaacatttgattcagagt<br>tttttaatttgtactcgctgcaaaaaacggtacgctttgagttaaaacccgtgggagaaaccgcg<br>tcatttgtggaagactttaaaaacgagggcttgaaacgtgttgtgagcgaagatgaaaggcgagc<br>cgtcgattaccagaaagttaaggaaataattgacgattaccatcgggatttcattgaagaaagtt<br>taaattattttccggaacaggtgagtaaagatgctcttgagcaggcgtttcatctttatcagaaa<br>ctgaaggcagcaaaagttgaggaaagggaaaaagcgctgaaagaatgggaagcgctgcagaaaaa<br>gctacgtgaaaaagtggtgaaatgcttctcggactcgaataaagcccgcttctcaaggattgata<br>aaaaggaactgattaaggaagacctgataaattggttggtcgcccagaatcgcgaggatgatatc<br>cctacggtcgaaacgtttaacaacttcaccacatattttaccggcttccatgagaatcgtaaaaa<br>tatttactccaaagatgatcacgccaccgctattagctttcgccttattcatgaaaatcttccaa<br>agtttttgacaacgtgattagcttcaataagttgaaagagggtttccctgaattaaaatttgat<br>aaagtgaaagaggatttagaagtagattatgatctgaagcatgcgtttgaaatagaatatttcgt<br>taacttcgtgacccaagcgggcatagatcagtataattatctgttaggagggaaaaccctggagg<br>acgggacgaaaaaacaagggatgaatgagcaaattaatctgttcaaacaacagcaaacgcgagat<br>aaagcgcgtcagattcccaaactgatcccctgttcaaacagattcttagcgaaaggactgaaag<br>ccagtcctttattcctaaacaatttgaaagtgatcaggagttgttcgattcactgcagaagttac<br>ataataactgccaggataaattcaccgtgctgcaacaagccattctcggtctggcagaggcggat<br>cttaagaaggtcttcatcaaaacctctgatttaaatgccttatctaacaccattttcgggaatta<br>cagcgtcttttccgatgcactgaacctgtataaagaaagcctgaaaacgaaaaaagcgcaggagg<br>cttttgagaaactaccggcccattctattcacgacctcattcaatacttggaacagttcaattcc<br>agcctggacgcggaaaaacaacagagcaccgacaccgtcctgaactacttcatcaagaccgatga<br>attatattctcgcttcattaaatccactagcgaggctttcactcaggtgcagcctttgttcgaac<br>tggaagccctgtcatctaagcgccgcccaccggaatcggaagatgaaggggcaaaagggcaggaa<br>ggcttcgagcagatcaagcgtattaaagcttacctggatacgcttatggaagcggtacactttgc<br>aaagccgttgtatcttgttaagggtcgtaaaatgatcgaagggctcgataaagaccagtcctttt<br>atgaagcgtttgaaatggcgtaccaagaacttgaatcgttaatcattcctatctataacaaagcg<br>cggagctatctgtcgcggaaacctttcaaggccgataaattcaagattaattttgacaacaacac<br>gctactgagcggatgggatgcgaacaaggaaactgctaacgcgtccattctgtttaagaaagacg<br>ggttatattaccttggaattatgccgaaaggtaagacctttctctttgactactttgtatcgagc

CA_Cpf1_3	AAGCATTGGCCGTAAGTGCGATTCCGGAAAGGAGATATACatgcatacaggcggtcttcttagta<br>tggacgcgaaagagttcacaggtcagtatccgttgtcgaaaacattacgattcgaacttcggccc<br>atcggccgcacgtgggataacctggaggcctcaggctacttagcggaagaccgccatcgtgccga<br>atgttatcctcgtgcgaaagagttattggatgacaaccatcgtgccttcctgaatcgtgtgttgc<br>cacaaatcgatatggattggcacccgattgcggaggccttttgtaaggtacataaaaaccctggt<br>aataaagaacttgcccaggattacaaccttcagttgtcaaagcgccgtaaggagatcagcgcata<br>tcttcaggatgcagatggctataaaggcctgttcgcgaagcccgccttagacgaagctatgaaaa<br>ttgcgaaagaaaacgggaacgaaagtgatattgaggttctcgaagcgtttaacggttttagcgta<br>tacttcaccggttatcatgagtcacgcgagaacatttatagcgatgaggatatggtgagcgtagc<br>ctaccgaattactgaggataatttcccgcgctttgtctcaaacgctttgatctttgataaattaa<br>acgaaagccatccggatattatctctgaagtatcgggcaatcttggagttgatgacattggtaag<br>tactttgacgtgtcgaactataacaattttctttcccaggccggtatagatgactacaatcacat<br>tattggcggccatacaaccgaagacggactgatacaagcgtttaatgtcgtattgaacttacgtc<br>accaaaaagaccctggctttgaaaaaattcagttcaaacagctctacaaacaaatcctgagcgtg<br>cgtaccagcaaaagctacatcccgaaacagtttgacaactctaaggagatggttgactgcatttg<br>cgattatgtcagcaaaatagagaaatccgaaacagtagaacgggccctgaaactagtccgtaata<br>tcagttctttcgacttgcgcgggatctttgtcaataaaaagaacttgcgcatactgagcaacaaa<br>ctgataggagattgggacgcgatcgaaaccgcattgatgcatagttcttcatcagaaaacgataa<br>gaaaagcgtatatgatagcgcggaggcttttacgttggatgacatcttttcaagcgtgaaaaaat<br>tttctgatgcctctgccgaagatattggcaacagggcggaagacatctgtagagtgataagtgag<br>acggcccctttttatcaacgatctgcgagcggtggacctggatagcctgaacgacgatggttatga<br>agcggccgtctcaaaaattcgggagtcgctggagccttatatggatcttttccatgaactggaaa<br>ttttctcggttggcgatgagttcccaaaatgcgcagcattttacagcgaactggaggaagtcagc<br>gaacagctgatcgaaattattccgttattcaacaaggcgcgttcgttctgcacccggaaacgcta<br>tagcaccgataagattaaagtgaacttaaaattcccgaccttggcggacgggtgggacctgaaca<br>aagagagagacaacaaagccgcgattctgcggaaagacggtaagtattatctggcaattctggat<br>atgaagaaagatctgtcaagcattaggaccagcgacgaagatgaatccagcttcgaaaagatgga<br>gtataaactgttaccgagtccagtaaaaatgctgccaaagatattcgtaaaatcgaaagccgcta<br>aggaaaaatatggcctgacagatcgtatgcttgaatgctacgataaaggtatgcataagtcgggt

PC_Cpf1_3	AAGCATTGGCCGTAAGTGCGATTCCGGAAAGGAGATATACatggatagtttgaaagatttcacca<br>atctgtaccctgtcagtaagacattgagatttgaattaaagcccgttggaaagactttagaaaat<br>atcgagaaagcaggtattttgaaagaggatgagcatcgtgcagaaagttatcggagggtgaagaa<br>aataattgatacttatcataaggtatttatcgattcttctcttgaaaatatggctaaaatgggta<br>ttgagaatgaaataaaagcaatgctccaaagtttctgcgaattgtataaaaaagatcatcgcact<br>gagggtgaagacaaggcattagataaaattcgagcagtacttcgtggcctgattgttggggcttt<br>cactggtgtttgcggaagacgggaaaatacagtccaaaacgagaagtacgagagtttgttcaaag<br>aaaagttgataaaagaaattttacctgattttgtgctctctactgaggctgaaagcttgcctttc<br>tctgttgaagaagctacgaggtcactgaaggagtttgatagctttacatcctactttgctggttt<br>ttacgagaatagaaagaatatatactcgacgaaacctcaatccactgccattgcttatcgtctta<br>ttcatgagaacttgccgaagttcattgataatattcttgtttttcagaagatcaaagagcctata<br>gccaaagagctggaacatattcgtgcggacttttctgccgggggtacataaaaaaggatgagag<br>attggaggatatttttttcgttgaactattatatccacgtgttatctcaggctgggatcgaaaaat<br>ataacgcattgattgggaagattgtgacagaaggagatggagagatgaaagggctcaatgaacac<br>atcaacctttacaaccaacaaagaggcagagaggatcggctccctctttttaggcctctttataa<br>acagatattgagtgacagagagcaattatcatacttgcctgagagttttgaaaaagatgaggagc<br>tcctcagggctctaaaagagttctatgatcatatcgcagaagacattctcggacgtactcaacag<br>ttgatgacttctatttcagaatatgatttatctcggatatacgtaaggaacgatagccaattgac<br>tgatatatcaaaaaaaatgttgggagattggaatgctatctacatggctagagaacgagcatatg<br>accacgagcaggctcccaaaagaatcacggcgaaatacgagagggacaggattaaagctcttaaa<br>ggagaagagagtataagtctggcaaatcttaatagttgtattgcctttctggacaatgttagaga<br>ttgccgtgtagatacttatctttccacactgggccagaaggaaggaccacatggtctatctaatc<br>tcgttgagaacgtttttgcctcataccatgaagcagagcaattgttgagctttccataccccgaa<br>gagaataatctgattcaggacaaggacaatgtggtgttaattaagaatcttctcgacaatatcag<br>tgatctgcagaggttcttgaaacctctttggggtatgggagacgaacccgataaagatgaaagat<br>tttatggagagtataattatatccgaggagctctagatcaggtgatccctctgtacaataaggta<br>aggaactacctcactcggaagccttattcgaccagaaaagtaaaactcaattttgggaattctca<br>attgcttagtggttgggatagaaataaggaaaaggataatagctgtgtgattttgcgtaaggggc<br>agaacttctatttggctattatgaacaataggcacaaaagaagtttcgaaaacaaggtgttgccc

FB_Cpf1_3	AAGCATTGGCCGTAAGTGCGATTCCGGAAAGGAGATATACatgaccaataaattcactaaccagt<br>attctctctctaagaccctgcgctttgaactgattccgcaggggaaaaccttggagttcattcaa<br>gaaaaaggcctcttgtctcaggataaacagagggctgaatcttaccaagaaatgaagaaaactat<br>tgataagtttcataaatatttcattgatttagccttgtctaacgccaaattaactcacttggaaa<br>cgtatctggagttatacaacaaatctgccgaaactaagaaagaacagaaatttaaagacgatttg<br>aaaaaagtacaggacaatctgcgtaaagaaattgtcaaatccttcagtgacggcgatgctaaaag<br>catttttgccattctggacaaaaaagagttgattactgtggaattagaaaagtggtttgaaaaca<br>atgagcagaaagacatctacttcgatgagaaattcaaaactttcaccacctattttacaggattt<br>catcaaaaccggaagaacatgtactcagtagaaccgaactccacggccattgcgtatcgtttgat<br>ccatgagaatctgcctaaatttctggagaatgcgaaagcctttgaaaagattaagcaggtcgaat<br>cgctgcaagtgaattttcgtgaactcatgggcgaatttggtgacgaaggtctaatcttcgttaac<br>gaactggaagaaatgtttcagattaattactacaatgacgtgctatcgcagaacggtatcacaat<br>ctacaatagtattatctcagggttcacaaaaaacgatataaaatacaaaggcctgaacgagtata<br>tcaataactacaaccaaacaaaggacaaaaaggataggcttccgaaactgaagcagttatacaaa<br>cagattttatctgacagaatctccctgagctttctgccggatgctttcactgatgggaagcaggt<br>tctgaaagcgattttcgatttttataagattaacttactgagctacacgattgaaggtcaagaag<br>aatctcaaaacttactgctcttgatccgtcaaaccattgaaaatctatcatcgttcgatacgcag<br>aaaatctacctcaaaaacgatactcacctgactacgatctctcagcaggttttcggggattttag<br>tgtattttcaacagctctgaactactggtatgaaaccaaagtcaatccgaaattcgagacggaat<br>attctaaggccaacgaaaaaaaacgtgagattcttgataaagctaaagccgtatttactaaacag<br>gattacttttctattgctttcctgcaggaagttttatcggagtatatcctgaccctggatcatac<br>atctgatatcgttaaaaaacacagcagcaattgcatcgctgactatttcaaaaaccactttgtcg<br>ccaaaaaagaaaacgaaacagacaagactttcgatttcattgctaacatcaccgcaaaataccag<br>tgtattcagggtatcttggaaaacgccgaccaatacgaagacgaactgaaacaagatcagaagct<br>gatcgataatttaaaattcttcttagatgcaatcctggagctgctgcacttcatcaaaccgcttc<br>atttaaagagcgagtccattaccgaaaaggacaccgccttctatgacgtttttgaaaattattat<br>gaagccctctccttgctgactccgctgtataatatggtacgcaattacgtaacccagaaaccata<br>ttctaccgaaaaaattaaactgaactttgaaaacgcacagctgctcaacggttgggacgcgaata<br>aagaaggtgactacctcaccaccatcctgaaaaaagatggtaactattttctggcaattatggat

CR_Cpf1_3	AAGCATTGGCCGTAAGTGCGATTCCGGAAAGGAGATATACatgtctttcgactctttcaccaacc<br>tgtactctctgtctaaaaccctgaaattcgaaatgcgtccggttggtaacacccagaaaatgctg<br>gacaacgcgggtgttttcgaaaaagacaaactgatccagaaaaaatacggtaaaaccaaaccgta<br>cttcgaccgtctgcaccgtgaattcatcgaagaagcgctgaccggtgttgaactgatcggtctgg<br>acgaaaacttccgtaccctggttgactggcagaaagacaaaaaaaacaacgttgcgatgaaagcg<br>tacgaaaactctctgcagcgtctgcgtaccgaaatcggtaaaatcttcaacctgaaagcggaaga<br>ctgggttaaaaacaaatacccgatcctgggtctgaaaaacaaaaacaccgacatcctgttcgaag<br>aagcggttttcggtatcctgaaagcgcgttacggtgaagaaaaagacaccttcatcgaagttgaa<br>gaaatcgacaaaaccggtaaatctaaaatcaaccagatctctatcttcgactcttggaaaggttt<br>caccggttacttcaaaaaattcttcgaaacccgtaaaaacttctacaaaaacgacggtacctcta<br>ccgcgatcgcgacccgtatcatcgaccagaacctgaaacgtttcatcgacaacctgtctatcgtt<br>gaatctgttcgtcagaaagttgacctggcggaaaccgaaaaatctttctctatctctctgtctca<br>gttcttctctatcgacttctacaacaaatgcctgctgcaggacggtatcgactactacaacaaaa<br>tcatcggtggtgaaaccctgaaaaacggtgaaaaactgatcggtctgaacgaactgatcaaccag<br>taccgtcagaacaacaaagaccagaaaatcccgttcttcaaactgctggacaaacagatcctgtc<br>tgaaaaaatcctgttcctggacgaaatcaaaaacgacaccgaactgatcgaagcgctgtctcagt<br>tcgcgaaaaccgcggaagaaaaaaccaaaatcgttaaaaaactgttcgcggacttcgttgaaaac<br>aactctaaatacgacctggcgcagatctacatctctcaggaagcgttcaacaccatctctaacaa<br>atggacctctgaaaccgaaaccttcgcgaaatacctgttcgaagcgatgaaatctggtaaactgg<br>cgaaatacgaaaaaaaagacaactcttacaaattcccggacttcatcgcgctgtctcagatgaaa<br>tctgcgctgctgtctatctctctggaaggtcacttctggaaagaaaaatactacaaaatctctaa<br>attccaggaaaaaccaactgggaacagttcctggcgatcttcctgtacgaattcaactctctgt<br>tctctgacaaaatcaacaccaaagacggtgaaaccaaacaggttggttactacctgttcgcgaaa<br>gacctgcacaacctgatcctgtctgaacagatcgacatcccgaaagactctaaagttaccatcaa<br>agacttcgcggactctgttctgaccatctaccagatggcgaaatacttcgcggttgaaaaaaaac<br>gtgcgtggctggcggaatacgaactggactctttctacacccagccggacaccggttacctgcag<br>ttctacgacaacgcgtacgaagacatcgttcaggtttacaacaaactgcgtaactacctgaccaa<br>aaaaccgtactctgaagaaaaatggaaactgaacttcgaaaactctaccctggcgaacggttggg<br>acaaaaacaaagaatctgacaactctgcggttatcctgcagaaaggtggtaaatactacctgggt

SC_Cpf1_3	AAGCATTGGCCGTAAGTGCGATTCCGGAAAGGAGATATACatgacccagttcgaaggtttcacca<br>acctgtaccaggtttctaaaaccctgcgtttcgaactgatcccgcagggtaaaaccctgaaacac<br>atccaggaacagggtttcatcgaagaagacaaagcgcgtaacgaccactacaaagaactgaaacc<br>gatcatcgaccgtatctacaaaacctacgcggaccagtgcctgcagctggttcagctggactggg<br>aaaacctgtctgcggcgatcgactcttaccgtaaagaaaaaaccgaagaaacccgtaacgcgctg<br>atcgaagaacaggcgacctaccgtaacgcgatccacgactacttcatcggtcgtaccgacaacct<br>gaccgacgcgatcaacaaacgtcacgcggaaatctacaaaggtctgttcaaagcggaactgttca<br>acggtaaagttctgaaacagctgggtaccgttaccaccaccgaacacgaaaacgcgctgctgcgt<br>tctttcgacaaattcaccacctacttctctggtttctacgaaaaccgtaaaaacgttttctctgc<br>ggaagacatctctaccgcgatcccgcaccgtatcgttcaggacaacttcccgaaattcaaagaaa<br>actgccacatcttcacccgtctgatcaccgcggttccgtctctgcgtgaacacttcgaaaacgtt<br>aaaaaagcgatcggtatcttcgtttctacctctatcgaagaagttttctctttcccgttctacaa<br>ccagctgctgacccagacccagatcgacctgtacaaccagctgctgggtggtatctctcgtgaag<br>cgggtaccgaaaaaatcaaaggtctgaacgaagttctgaacctggcgatccagaaaaacgacgaa<br>accgcgcacatcatcgcgtctctgccgcaccgtttcatcccgctgttcaaacagatcctgtctga<br>ccgtaacaccctgtctttcatcctggaagaattcaaatctgacgaagaagttatccagtctttct<br>gcaaatacaaaaccctgctgcgtaacgaaaacgttctggaaaccgcggaagcgctgttcaacgaa<br>ctgaactctatcgacctgacccacatcttcatctctcacaaaaaactggaaaccatctcttctgc<br>gctgtgcgaccactgggacaccctgcgtaacgcgctgtacgaacgtcgtatctctgaactgaccg<br>gtaaaatcaccaaatctgcgaaagaaaaagttcagcgttctctgaaacacgaagacatcaacctg<br>caggaaatcatctctgcggcgggtaaagaactgtctgaagcgttcaaacagaaaacctctgaaat<br>cctgtctcacgcgcacgcggcgctggaccagccgctgccgaccaccctgaaaaaacaggaagaaa<br>aagaaatcctgaaatctcagctggactctctgctgggtctgtaccacctgctggactggttcgcg<br>gttgacgaatctaacgaagttgacccggaattctctgcgcgtctgaccggtatcaaactggaaat<br>ggaaccgtctctgtctttctacaacaaagcgcgtaactacgcgaccaaaaaaccgtactctgttg<br>aaaaattcaaactgaacttccagatgccgaccctggcgtctggttgggacgttaacaaagaaaaa<br>aacaacggtgcgatcctgttcgttaaaaacggtctgtactacctgggtatcatgccgaaacagaa<br>aggtcgttacaaagcgctgtctttcgaaccgaccgaaaaaacctctgaaggtttcgacaaaatgt<br>actacgactacttcccggacgcggcgaaaatgatcccgaaatgctctacccagctgaaagcggtt

SD_Cpf1_4	GTACTTATGAAGACATTTCCGGGTTTTATCGTGAGgtcgataatctgtgctacaaactggagttc<br>tgcccgattaaaacctcgtttatagaaaacctgatagataacggcgacctgtatctgtttcgcat<br>caataacaaagacttcagcagtaaatcgaccggcaccaagaaccttcatacgttatatttacaag<br>ctatattcgatgaacgtaatctgaacaatccgacaattatgctgaatgggggagcagaactgttc<br>tatcgtaaagaaagtattgagcagaaaaaccgtatcacacacaaagccggttcaattctcgtgaa<br>taaggtgtgtaaagacggtacaagcctggatgataagatacgtaatgaaatttatcaatatgaga<br>ataaatttattgataccctgtctgatgaagctaaaaaggtgttaccgaatgtcattaaaaaggaa<br>gctacccatgacattacaaaagataaacgtttcactagtgacaaattcttctttcactgcccccct<br>gacaattaattataaggaaggcgataccaagcagttcaataacgaagtgctgagttttctgcgtg<br>gaaatcctgacatcaacattatcggcattgaccgcggagagcgtaatttaatctatgtaacggtt<br>ataaaccagaaaggcgagattctggattcggtttcattcaataccgtgaccaacaagagttcaaa<br>aatcgagcagacagtcgattatgaagagaaattggcagtccgcgagaaagagaggattgaagcaa<br>aacgttcctgggactctatctcaaaaattgcgacactaaaggaaggttatctgagcgcaatagtt<br>cacgagatctgtctgttaatgattaaacacaacgcgatcgttgtcttagagaatcttaatgcagg<br>ctttaagcgtattcgtggcggtttatcagaaaaaagtgtttatcaaaaattcgaaaaaatgttga<br>ttaacaaactgaactattttgtcagcaagaaggaatccgactggaataaaccgtctggtctgctg<br>aatggactgcagctttcggatcagtttgaaagcttcgaaaaactgggtattcagtctggttttat<br>tttttacgtgccggctgcatatacctcaaagattgatccgaccacgggcttcgccaatgttctga<br>atctgtcgaaggtacgcaatgttgatgcgatcaaaagctttttttctaacttcaacgaaattagt<br>tatagcaagaaagaagcccttttcaaattctcattcgatctggattcactgagtaagaaaggctt<br>tagtagctttgtgaaatttagtaagagtaaatggaacgtctacacctttggagaacgtatcataa<br>agccaaagaataagcaaggttatcgggaggacaaaagaatcaacttgaccttcgagatgaagaag<br>ttacttaacgagtataaggtttcttttgatcttgaaaataacttgattccgaatctcacgagtgc<br>caacctgaaggatactttttggaaagagctattctttatcttcaagactacgctgcagctccgta<br>acagcgttactaacggtaaagaagatgtgctcatctctccggtcaaaaatgcgaagggtgaattc<br>ttcgtttcgggaacgcataacaagactcttccgcaagattgcgatgcgaacggtgcataccatat<br>tgcgttgaaaggtctgatgatactcgaacgtaacaaccttgtacgtgaggagaaagatacgaaaa<br>agattatggcgatttcaaacgtggattggttcgagtacgtgcagaaacgtagaggcgttctgtaa<br>GAAATCATCCTTAGCGAAAGCTAAGGCTGATACTCTTCC

CT_Cpf1_4	GTACTTATGAAGACATTTCCGGGTTTTATCGTGAGgttgaaaaacagggttaccgtatgcacttc<br>gaaaacatctctgcggaaaccatcgacgaatacgttgaaaaaggtgacctgttcctgttccagat<br>ctacaacaaagacttcgttaaagcggcgaccggtaaaaaagacatgcacaccatctactggaacg<br>cggcgttctctccggaaaacctgcaggacgttgttgttaaactgaacggtgaagcggaactgttc<br>taccgtgacaaatctgacatcaaagaaatcgttcaccgtgaaggtgaaatcctggttaaccgtac<br>ctacaacggtcgtaccccggttccggacaaaatccacaaaaaactgaccgactaccacaacggtc<br>gtaccaaagacctgggtgaagcgaaagaatacctggacaaagttcgttacttcaaagcgcactac<br>gacatcaccaaagaccgtcgttacctgaacgacaaaatctacttccacgttccgctgaccctgaa<br>cttcaaagcgaacggtaaaaaaaacctgaacaaaatggttatcgaaaaattcctgtctgacgaaa<br>aagcgcacatcatcggtatcgaccgtggtgaacgtaacctgctgtactactctatcatcgaccgt<br>tctggtaaaatcatcgaccagcagtctctgaacgttatcgacggtttcgactaccgtgaaaaact<br>gaaccagcgtgaaatcgaaatgaaagacgcgcgtcagtcttggaacgcgatcggtaaaatcaaag<br>acctgaaagaaggttacctgtctaaagcggttcacgaaatcaccaaaatggcgatccagtacaac<br>gcgatcgttgttatggaagaactgaactacggtttcaaacgtggtcgtttcaaagttgaaaaaca<br>gatctaccagaaattcgaaaacatgctgatcgacaaaatgaactacctggttttcaaagacgcgc<br>cggacgaatctccgggtggtgttctgaacgcgtaccagctgaccaacccgctggaatctttcgcg<br>aaactgggtaaacagaccggtatcctgttctacgttccggcggcgtacacctctaaaatcgaccc<br>gaccaccggtttcgttaacctgttcaacacctcttctaaaaccaacgcgcaggaacgtaaagaat<br>tcctgcagaaattcgaatctatctcttactctgcgaaagacggtggtatcttcgcgttcgcgttc<br>gactaccgtaaattcggtacctctaaaaccgaccacaaaaacgtttggaccgcgtacaccaacgg<br>tgaacgtatgcgttacatcaaagaaaaaaaacgtaacgaactgttcgacccgtctaaagaaatca<br>aagaagcgctgacctcttctggtatcaaatacgacggtggtcagaacatcctgccggacatcctg<br>cgttctaacaacaacggtctgatctacaccatgtactcttctttcatcgcggcgatccagatgcg<br>tgtttacgacggtaaagaagactacatcatctctccgatcaaaaactctaaaggtgaattcttcc<br>gtaccgacccgaaacgtcgtgaactgccgatcgacgcggacgcgaacggtgcgtacaacatcgcg<br>ctgcgtggtgaactgaccatgcgtgcgatcgcggaaaaattcgacccggactctgaaaaaatggc<br>gaaactggaactgaaacacaaagactggttcgaattcatgcagacccgtggtgactaaGAAATCA<br>TCCTTAGCGAAAGCTAAGGCTGATACTCTTCC

TX_Cpf1_4	GTACTTATGAAGACATTTCCGGGTTTTATCGTGAGgtagaaaaccagggttacgtaattagcttt<br>gacaaaatcaaagagacctatatacagagccaggtggaacagggtaatctctacttattccagat<br>ttataacaaggatttctcgccctacagcaaaggcaaaccaaacctgcatactctgtactggaaag<br>ccctgtttgaagaagcgaacctgaataacgtagtggcgaagttgaacggtgaagcggaaatcttc<br>ttccgtcgtcactccattaaggcctctgataaagttgtccatccggcaaatcaggccattgataa<br>taagaatccacacacggaaaaaacgcagtcaacctttgaatatgacctcgttaaagacaaacgct<br>acacgcaagataagttctttttccacgtcccaatcagcctcaactttaaagcacaaggggtttca<br>aagtttaatgataaagtcaatgggttcctcaagggcaacccggatgtcaacattataggtataga<br>caggggcgaacgccatctgctttactttaccgtagtgaatcagaaaggtgaaatactggttcagg<br>aatcattaaataccttgatgtcggacaaagggcacgttaatgattaccagcagaaactggataaa<br>aaagaacaggaacgtgatgctgcgcgtaaatcgtggaccacggttgagaacattaaagagctgaa<br>agaggggtatctaagccatgtggtacacaaactggcgcacctcatcattaaatataacgcaatag<br>tctgcctagaagacttgaattttggctttaaacgcggccgcttcaaagtggaaaaacaagtttat<br>caaaaatttgaaaaggcgcttatagataaactgaattatctggtttttaaagaaaaggaacttgg<br>tgaggtagggcactacttgacagcttatcaactgacggccccgttcgaatcattcaaaaaactgg<br>gcaaacagtctggcattctgttttacgtgccggcagattatacttcaaaaatcgatccaacaact<br>ggctttgtgaacttcctggacctgagatatcagtctgtagaaaaagctaaacaacttcttagcga<br>ttttaatgccattcgttttaacagcgttcagaattactttgaattcgaaattgactataaaaaac<br>ttactccgaaacgtaaagtcggaacccaaagtaaatgggtaatttgtacgtatggcgatgtcagg<br>tatcagaaccgtcggaatcaaaaaggtcattgggagaccgaagaagtgaacgtgaccgaaaagct<br>gaaggctctgttcgccagcgattcaaaaactacaactgtgatcgattacgcaaatgatgataacc<br>tgatagatgtgattttagagcaggataaagccagctttttttaaagaactgttgtggctcctgaaa<br>cttacgatgaccttacgacattccaagatcaaatcggaagatgattttattctgtcaccggtcaa<br>gaatgagcagggtgaattctatgatagtaggaaagccggcgaagtgtggccgaaagacgccgacg<br>ccaatggcgcctatcatatcgcgctcaaagggctttggaatttgcagcagattaaccagtgggaa<br>aaaggtaaaaccctgaatctggctatcaaaaaccaggattggtttagctttatccaagagaaacc<br>gtatcaggaatgaGAAATCATCCTTAGCGAAAGCTAAGGCTGATACTCTTCC

CA_Cpf1_4	GTACTTATGAAGACATTTCCGGGTTTTATCGTGAGgtggccggagccggttactatatgagtctg<br>agaaaaattccgtgcagcgaagtgtaccgtctgttagacgagaaatcgatttatctatttcaaat<br>ttataacaaagattactctgaaaatgcacatggtaataagaacatgcataccatgtactgggagg<br>gtctcttttccccgcaaaacctggagtcgcccgttttcaagttgtcgggtggggcagaacttttc<br>tttcgaaaatcctcaatccctaacgatgccaaaacagtacacccgaaaggctcagtgctggttcc<br>acgtaatgatgttaacggtcggcgtattccagattcaatctaccgcgaactgacacgctatttta<br>accgtggcgattgccgaatcagtgacgaagccaaaagttatcttgacaaggttaagactaaaaaa<br>gcggaccatgacattgtgaaagatcgccgctttaccgtggataaaatgatgttccacgtcccgat<br>tgcgatgaactttaaggcgatcagtaaaccgaacttaaacaaaaaagtcattgatggcatcattg<br>atgatcaggatctgaaaatcattggtattgatcgtggcgagcggaacttaatttacgtcacgatg<br>gttgacagaaaagggaatatcttatatcaggattctcttaacatcctcaatggctacgactatcg<br>taaagctctggatgtgcgcgaatatgacaacaaggaagcgcgtcgtaactggactaaagtggagg<br>gcattcgcaaaatgaaggaaggctatctgtcattagcggtctcgaaattagcggatatgattatc<br>gaaaataacgccatcatcgttatggaggacctgaaccacggattcaaagcgggccgctcaaagat<br>tgaaaaacaagtttatcagaaatttgagagtatgctgattaacaaactgggctatatggtgttaa<br>aagacaagtcaattgaccaatcaggtggcgcgctgcatggataccagctggcgaaccatgttacc<br>accttagcatcagttggaaagcagtgtggggttatcttttatataccggcagcgttcactagtaa<br>aatagatccgaccactggtttcgccgatctctttgccctgagtaacgttaaaaacgtagcgagca<br>tgcgtgaattcttttccaaaatgaaatctgtcatttatgataaagctgaaggcaaattcgcattc<br>acctttgattacttggattacaacgtgaagagcgaatgtggtcgtacgctgtggaccgtttacac<br>cgttggtgagcgcttcacctattcccgtgtgaaccgcgaatatgtacgtaaagtccccaccgata<br>ttatctatgatgccctccagaaagcaggcattagcgtcgaaggagacttaagggacagaattgcc<br>gaaagcgatggcgatacgctgaagtctatttttacgcattcaaatacgcgctagatatgcgcgt<br>tgagaatcgcgaggaagactacattcaatcacctgtgaaaaatgcctctggggaatttttttgtt<br>caaaaaatgctggtaaaagcctcccacaagatagcgatgcaaacggtgcatataacattgccctg<br>aaaggtattcttcaattacgcatgctgtctgagcagtacgaccccaacgcggaatctattagact<br>tccgctgataaccaataaagcctggctgacattcatgcagtctggcatgaagacctggaaaaatt<br>agGAAATCATCCTTAGCGAAAGCTAAGGCTGATACTCTTCC

PC_Cpf1_4	GTACTTATGAAGACATTTCCGGGTTTTATCGTGAGgttgaggatcaggggtataagctctctttc<br>cgaaaagtttcggaatcttatgtctattcattaatagatcaaggcaagttgtatttatttcagat<br>atacaacaaggacttttctccctgcagcaaagggacacctaatctgcataccttgtattggagaa<br>tgctttttgacgagcgcaatttggcagatgtcatatacaaactggatgggaaggctgaaatcttt<br>ttccgagagaagagtttgaaaaatgatcatcccacgcatccggctggtaagcctatcaaaaagaa<br>aagtcgacaaaaaaaaggagaggagagtctgtttgagtatgatttagtcaaggataggcactata<br>cgatggataagttccagtttcatgtgcctattactatgaattttaaatgttctgcaggaagcaaa<br>gtcaatgatatggttaatgctcatattcgagaggcaaaggatatgcatgtcattggaattgatcg<br>tggagaacgcaatctgctgtatatatgcgtgatagatagtcgagggacgattttggatcaaattt<br>ctctgaatacgattaacgatatagactatcatgatttattggagagtcgagacaaagaccgtcag<br>caggagcgccgaaactggcaaactatcgaagggatcaaggagctaaaacaaggctaccttagtca<br>ggcggttcatcggatagccgaactgatggtggcttataaggctgtagttgctttggaggatttga<br>atatggggttcaaacgtgggcggcagaaagtagaaagttctgtttatcagcagtttgagaaacag<br>ctgatagataagctcaactatcttgtggacaagaagaaaaggcctgaagatattggaggattgtt<br>gagagcctatcaatttacggccccatttaagagttttaaggaaatgggaaagcaaaacggcttct<br>tgttttatatcccggcttggaacacgagcaacatagatccgactactggatttgttaatttattt<br>catgcccagtatgaaaatgtagataaagcgaagagcttctttcaaaagtttgattcaattagtta<br>caacccgaagaaagactggtttgagtttgcattcgattataaaaactttactaaaaaggctgaag<br>gaagtcgttctatgtggatattatgcacacatggttcccgaataaagaattttagaaattcccag<br>aagaatggtcaatgggattccgaagaattcgccttgacggaggcttttaagtctctttttgtgcg<br>atatgagatagattataccgctgatttgaaaacagctattgtggacgaaaagcaaaaagacttct<br>tcgtggatcttctgaagctattcaaattgacagtacagatgcgcaacagctggaaagagaaggat<br>ttggattatctaatctctcctgtagcaggggctgatggccgtttcttcgatacaagagagggaaa<br>taaaagtctgcctaaggatgcagatgccaatggagcttataatattgccctaaaaggactttggg<br>ctctacgccagattcggcaaacttcagaaggcggtaaactcaaattggcgatttccaataaggaa<br>tggctacagtttgtgcaagagagatcttacgagaaagactgaGAAATCATCCTTAGCGAAAGCTA<br>AGGCTGATACTCTTCC

FB_Cpf1_4	GTACTTATGAAGACATTTCCGGGTTTTATCGTGAGgtagaacaccaaggctataaaattaacttc<br>aaaaacatcgacagcgaatacatcgacggtttagttaacgagggcaaactgtttctgttccagat<br>ctattcaaaggattttagcccgttctctaaaggcaaaccaaatatgcatacgttgtactggaaag<br>cactgtttgaagagcaaaacctgcagaatgtgatttataaactgaacggccaagctgagatttt t<br>ttccgtaaagcctcgattaaaccgaaaaatatcatccttcataagaagaaaataaagatcgctaa<br>aaaacacttcatagataaaaaaaccaaaacctccgaaatagtgcctgttcaaacaattaagaact<br>tgaatatgtactaccagggcaagatatcggaaaaggagttgactcaagacgatcttcgctatatc<br>gataacttttcgatttttaacgaaaaaaacaagacgatcgacatcatcaaagataaacgcttcac<br>tgtagataagttccagtttcatgtgccgattactatgaacttcaaagctaccgggggtagctata<br>tcaaccaaacggtgttggaatacctgcagaataacccggaagtcaaaatcattgggctggaccgc<br>ggagaacgtcaccttgtgtacttgaccttaatcgatcagcaaggcaacatcttaaaacaagaatc<br>gctgaataccattacggattcaaagattagcaccccgtatcataagctgctcgataacaaggaga<br>atgagcgcgacctggcccgtaaaaactggggcacggtggaaaacattaaggagttaaaggagggt<br>tatatttcccaggtagtgcataagatcgccactctcatgctcgaggaaaatgcgatcgttgtcat<br>ggaagacttaaacttcggatttaaacgtgggcgatttaaagtagagaaacaaatctaccagaagt<br>tagaaaaaatgctgattgacaaattaaattacttggtcctaaaagacaaacagccgcaagaattg<br>ggtggattatacaacgccctccaacttaccaataaattcgaaagttttcagaaaatgggtaaaca<br>gtcaggctttctttttttatgttcctgcgtggaacacatccaaaatcgaccctacaaccggcttcg<br>tcaattacttctatactaaatatgaaaacgtcgacaaagcaaaagcattctttgaaaagttcgaa<br>gcaatacgttttaacgctgagaaaaaatatttcgagttcgaagtcaagaaatactcagactttaa<br>ccccaaagctgagggcacacagcaagcgtggacaatctgcacctacggcgagcgcatcgaaacga<br>agcgtcaaaaagatcagaataacaaatttgtttcaacacctatcaacctgaccgagaagattgaa<br>gacttcttaggtaaaaatcagattgtttatggcgacggtaactgtataaaatctcaaatagcctc<br>aaaggatgataaagcatttttcgaaacattattatattggttcaaaatgacactgcagatgcgca<br>atagtgagacgcgtacagatattgattatcttatcagcccggtcatgaacgacaacggtactttt<br>tacaactccagagactatgaaaaacttgagaatccaactctccccaaagatgctgatgcgaacgg<br>tgcttatcacatcgcgaaaaaaggtctgatgctgctgaacaaaatcgaccaagccgatctgacta<br>agaaagttgacctaagcatttcaaatcgggactggttacagtttgttcaaaagaacaaatgaGAA<br>ATCATCCTTAGCGAAAGCTAAGGCTGATACTCTTCC

CR_Cpf1_4	GTACTTATGAAGACATTTCCGGGTTTTATCGTGAGgttgcggaagacggttaccgtatcgacttc<br>cagggtatctctgaccagtacatccacgaaaaaaacgaaaaaggtgaactgcacctgttcgaaat<br>ccacaacaaagactggaacctggacaaagcgcgtgacggtaaatctaaaaccacccagaaaaacc<br>tgcacaccctgtacttcgaatctctgttctctaacgacaacgttgttcagaacttcccgatcaaa<br>ctgaacggtcaggcggaaatcttctaccgtccgaaaaccgaaaaagacaaactggaatctaaaaa<br>agacaaaaaaggtaacaaagttatcgaccacaaacgttactctgaaaacaaaatcttcttccacg<br>ttccgctgaccctgaaccgtaccaaaaacgactcttaccgtttcaacgcgcagatcaacaacttc<br>ctggcgaacaacaaagacatcaacatcatcggtgttgaccgtggtgaaaaacacctggtttacta<br>ctctgttatcacccaggcgtctgacatcctggaatctggttctctgaacgaactgaacggtgtta<br>actacgcggaaaaactgggtaaaaaagcggaaaaccgtgaacaggcgcgtcgtgactggcaggac<br>gttcagggtatcaaagacctgaaaaaaggttacatctctcaggttgttcgtaaactggcggacct<br>ggcgatcaaacacaacgcgatcatcatcctggaagacctgaacatgcgtttcaaacaggttcgtg<br>gtggtatcgaaaaatctatctaccagcagctggaaaaagcgctgatcgacaaactgtctttcctg<br>gttgacaaaggtgaaaaaaacccggaacaggcgggtcacctgctgaaagcgtaccagctgtctgc<br>gccgttcgaaaccttccagaaaatgggtaaacagaccggtatcatcttctacacccaggcgtctt<br>acacctctaaatctgacccggttaccggttggcgtccgcacctgtacctgaaatacttctctgcg<br>aaaaaagcgaaagacgacatcgcgaaattcaccaaaatcgaattcgttaacgaccgtttcgaact<br>gacctacgacatcaaagacttccagcaggcgaaagaatacccgaacaaaaccgtttggaaagttt<br>gctctaacgttgaacgtttccgttgggacaaaaacctgaaccagaacaaaggtggttacacccac<br>tacaccaacatcaccgaaaacatccaggaactgttcaccaaatacggtatcgacatcaccaaaga<br>cctgctgacccagatctctaccatcgacgaaaaacagaacacctctttcttccgtgacttcatct<br>tctacttcaacctgatctgccagatccgtaacaccgacgactctgaaatcgcgaaaaaaaacggt<br>aaagacgacttcatcctgtctccggttgaaccgttcttcgactctcgtaaagacaacggtaacaa<br>actgccggaaaacggtgacgacaacggtgcgtacaacatcgcgcgtaaaggtatcgttatcctga<br>acaaaatctctcagtactctgaaaaaaacgaaaactgcgaaaaaatgaaatggggtgacctgtac<br>gtttctaacatcgactgggacaacttcgtttgaGAAATCATCCTTAGCGAAAGCTAAGGCTGATA<br>CTCTTCC

SC_Cpf1_4	GTACTTATGAAGACATTTCCGGGTTTTATCGTGAGctgaacccgctgctgtaccacatctctttc<br>cagcgtatcgcggaaaaagaaatcatggacgcggttgaaaccggtaaactgtacctgttccagat<br>ctacaacaaagacttcgcgaaaggtcaccacggtaaaccgaacctgcacaccctgtactggaccg<br>gtctgttctctccggaaaacctggcgaaaacctctatcaaactgaacggtcaggcggaactgttc<br>taccgtccgaaatctcgtatgaaacgtatggcgcaccgtctgggtgaaaaaatgctgaacaaaaa<br>actgaaagaccagaaaaccccgatcccggacaccctgtaccaggaactgtacgactacgttaacc<br>accgtctgtctcacgacctgtctgacgaagcgcgtgcgctgctgccgaacgttatcaccaaagaa<br>gtttctcacgaaatcatcaaagaccgtcgtttcacctctgacaaattcttcttccacgttccgat<br>caccctgaactaccaggcggcgaactctccgtctaaattcaaccagcgtgttaacgcgtacctga<br>aagaacacccggaaaccccgatcatcggtatcgaccgtggtgaacgtaacctgatctacatcacc<br>gttatcgactctaccggtaaaatcctggaacagcgttctctgaacaccatccagcagttcgacta<br>ccagaaaaaactggacaaccgtgaaaaagaacgtgttgcggcgcgtcaggcgtggtctgttgttg<br>gtaccatcaaagacctgaaacagggttacctgtctcaggttatccacgaaatcgttgacctgatg<br>atccactaccaggcggttgttgttctggaaaacctgaacttcggtttcaaatctaaacgtaccgg<br>tatcgcggaaaaagcggtttaccagcagttcgaaaaaatgctgatcgacaaactgaactgcctgg<br>ttctgaaagactacccggcggaaaaagttggtggtgttctgaacccgtaccagctgaccgaccag<br>ttcacctctttcgcgaaaatgggtacccagtctggtttcctgttctacgttccggcgccgtacac<br>ctctaaaatcgacccgctgaccggtttcgttgacccgttcgtttggaaaaccatcaaaaaccacg<br>aatctcgtaaacacttcctggaaggtttcgacttcctgcactacgacgttaaaaccggtgacttc<br>atcctgcacttcaaaatgaaccgtaacctgtctttccagcgtggtctgccgggtttcatgccggc<br>gtgggacatcgttttcgaaaaaaacgaaacccagttcgacgcgaaaggtaccccgttcatcgcgg<br>gtaaacgtatcgttccggttatcgaaaaccaccgtttcaccggtcgttaccgtgacctgtacccg<br>gcgaacgaactgatcgcgctgctggaagaaaaaggtatcgttttccgtgacggttctaacatcct<br>gccgaaactgctggaaaacgacgactctcacgcgatcgacaccatggttgcgctgatccgttctg<br>ttctgcagatgcgtaactctaacgcggcgaccggtgaagactacatcaactctccggttcgtgac<br>ctgaacggtgtttgcttcgactctcgtttccagaacccggaatggccgatggacgcggacgcgaa<br>cggtgcgtaccacatcgcgctgaaaggtcagctgctgctgaaccacctgaaagaatctaaagacc<br>tgaaactgcagaacggtatctctaaccaggactggctggcgtacatccaggaactgcgtaactat<br>aaGAAATCATCCTTAGCGAAAGCTAAGGCTGATACTCTTCC

SD_Cpf1_5	CGTATGATAAATACTTCCTTCATATGCCTATTACGATCaattataaggaaggcgataccaagcagttcaataacgaagtgctgagttttctgcgtggaaatcctgacatcaacattatcggcattgaccgcggagagcgtaatttaatctatgtaacggttataaaccagaaaggcgagattctggattcggtttcattcaataccgtgaccaacaagagttcaaaaatcgagcagacagtcgattatgaagagaaattggcagtccgcgagaaagagaggattgaagcaaaacgttcctgggactctatctcaaaaattgcgacactaaaggaaggttatctgagcgcaatagttcacgagatctgtctgttaatgattaaacacaacgcgatcgttgtcttagagaatcttaatgcaggctttaagcgtattcgtggcggtttatcagaaaaaagtgtttatcaaaaattcgaaaaaatgttgattaacaaactgaactattttgtcagcaagaaggaatccgactggaataaaccgtctggtctgctgaatggactgcagctttcggatcagtttgaaagcttcgaaaaactgggtattcagtctggttttattttttacgtgccggctgcatatacctcaaagattgatccgaccacgggcttcgccaatgttctgaatctgtcgaaggtacgcaatgttgatgcgatcaaaagctttttttctaacttcaacgaaattagttatagcaagaaagaagcccttttcaaattctcattcgatctggattcactgagtaagaaaggctttagtagctttgtgaaatttagtaagagtaaatggaacgtctacacctttggagaacgtatcataaagccaaagaataagcaaggttatcgggaggacaaaagaatcaacttgaccttcgagatgaagaagttacttaacgagtataaggtttcttttgatcttgaaaataacttgattccgaatctcacgagtgccaacctgaaggatacttttttggaaagagctattctttatcttcaagactacgctgcagctccgtaacagcgttactaacggtaaagaagatgtgctcatctctccggtcaaaaatgcgaagggtgaattcttcgtttcgggaacgcataacaagactcttccgcaagattgcgatgcgaacggtgcataccatattgcgttgaaaggtctgatgatactcgaacgtaacaaccttgtacgtgaggagaaagatacgaaaaagattatggcgatttcaaacgtggattggttcgagtacgtgcagaaacgtagaggcgttctgtaaGAAATCATCCTTAGCGAAAGCTAAGGCTGATACTCTTCC
CT_Cpf1_5	CGTATGATAAATACTTCCTTCATATGCCTATTACGATCaacttcaaagcgaacggtaaaaaaaacctgaacaaaatggttatcgaaaaattcctgtctgacgaaaaagcgcacatcatcggtatcgaccgtggtgaacgtaacctgctgtactactctatcatcgaccgttctggtaaaatcatcgaccagcagtctctgaacgttatcgacggtttcgactaccgtgaaaaactgaaccagcgtgaaatcgaaatgaaagacgcgcgtcagtcttggaacgcgatcggtaaaatcaaagacctgaaagaaggttacctgtctaaagcggttcacgaaatcaccaaaatggcgatccagtacaacgcgatcgttgttatggaagaactgaactacggtttcaaacgtggtcgtttcaaagttgaaaaacagatctaccagaaattcgaaaacatgctgatcgacaaaatgaactacctggttttcaaagacgcgccggacgaatctccgggtggtgttctgaacgcgtaccagctgaccaacccgctggaatctttcgcgaaactgggtaaacagaccggtatcctgttctacgttccggcggcgtacacctctaaaatcgacccgaccaccggtttcgttaacctgttcaacacctcttctaaaaccaacgcgcaggaacgtaaagaattcctgcagaaattcgaatctatctcttactctgcgaaagacggtggtatcttcgcgttcgcgttcgactaccgtaaattcggtacctctaaaaccgaccacaaaaacgtttggaccgcgtacaccaacggtgaacgtatgcgttacatcaaagaaaaaaaacgtaacgaactgttcgacccgtctaaagaaatcaaagaagcgctgacctcttctggtatcaaatacgacggtggtcagaacatcctgccggacatcctgcgttctaacaacaacggtctgatctacaccatgtactcttctttcatcgcggcgatccagatgcgtgtttacgacggtaaagaagactacatcatctctccgatcaaaaactctaaaggtgaattcttccgtaccgacccgaaacgtcgtgaactgccgatcgacgcggacgcgaacggtgcgtacaacatcgcgctgcgtggtgaactgaccatgcgtgcgatcgcggaaaaattcgacccggactctgaaaaaatggcgaaactggaactgaaacacaaagactggttcgaattcatgcagacccgtggtgactaaGAAATCATCCTTAGCGAAAGCTAAGGCTGATACTCTTCC

TX_Cpf1_5	CGTATGATAAATACTTCCTTCATATGCCTATTACGATCaactttaaagcacaaggggtttcaaagtttaatgataaagtcaatgggttcctcaagggcaacccggatgtcaacattataggtatagacaggggcgaacgccatctgctttactttaccgtagtgaatcagaaaggtgaaatactggttcaggaatcattaaataccttgatgtcggacaaagggcacgttaatgattaccagcagaaactggataaaaaagaacaggaacgtgatgctgcgcgtaaatcgtggaccacggttgagaacattaaagagctgaaagaggggtatctaagccatgtggtacacaaactggcgcacctcatcattaaatataacgcaatagtctgcctagaagacttgaattttggctttaaacgcggccgcttcaaagtggaaaaacaagtttatcaaaaatttgaaaaggcgcttatagataaactgaattatctggtttttaaagaaaaggaacttggtgaggtagggcactacttgacagcttatcaactgacggccccgttcgaatcattcaaaaaactgggcaaacagtctggcattctgttttacgtgccggcagattatacttcaaaaatcgatccaacaactggctttgtgaacttcctggacctgagatatcagtctgtagaaaaagctaaacaacttcttagcgattttaatgccattcgttttaacagcgttcagaattactttgaattcgaaattgactataaaaaacttactccgaaacgtaaagtcggaacccaaagtaaatgggtaatttgtacgtatggcgatgtcaggtatcagaaccgtcggaatcaaaaaggtcattgggagaccgaagaagtgaacgtgaccgaaaagctgaaggctctgttcgccagcgattcaaaaactacaactgtgatcgattacgcaaatgatgataacctgatagatgtgattttagagcaggataaagccagctttttttaaagaactgttgtggctcctgaaacttacgatgaccttacgacattccaagatcaaatcggaagatgattttattctgtcaccggtcaagaatgagcagggtgaattctatgatagtaggaaagccggcgaagtgtggccgaaagacgccgacgccaatggcgcctatcatatcgcgctcaaagggctttggaatttgcagcagattaaccagtgggaaaaaggtaaaaccctgaatctggctatcaaaaaccaggattggtttagctttatccaagagaaaccgtatcaggaatgaGAAATCATCCTTAGCGAAAGCTAAGGCTGATACTCTTCC
CA_Cpf1_5	CGTATGATAAATACTTCCTTCATATGCCTATTACGATCaactttaaggcgatcagtaaaccgaacttaaacaaaaaagtcattgatggcatcattgatgatcaggatctgaaaatcattggtattgatcgtggcgagcggaacttaatttacgtcacgatggttgacagaaaagggaatatcttatatcaggattctcttaacatcctcaatggctacgactatcgtaaagctctggatgtgcgcgaatatgacaacaaggaagcgcgtcgtaactggactaaagtggagggcattcgcaaaatgaaggaaggctatctgtcattagcggtctcgaaattagcggatatgattatcgaaaataacgccatcatcgttatggaggacctgaaccacggattcaaagcgggccgctcaaagattgaaaaacaagtttatcagaaatttgagagtatgctgattaacaaactgggctatatggtgttaaaagacaagtcaattgaccaatcaggtggcgcgctgcatggataccagctggcgaaccatgttaccaccttagcatcagttggaaagcagtgtggggttatcttttatataccggcagcgttcactagtaaaatagatccgaccactggtttcgccgatctctttgccctgagtaacgttaaaaacgtagcgagcatgcgtgaattcttttccaaaatgaaatctgtcatttatgataaagctgaaggcaaattcgcattcacctttgattacttggattacaacgtgaagagcgaatgtggtcgtacgctgtggaccgtttacaccgttggtgagcgcttcacctattcccgtgtgaaccgcgaatatgtacgtaaagtccccaccgatattatctatgatgccctccagaaagcaggcattagcgtcgaaggagacttaagggacagaattgccgaaagcgatggcgatacgctgaagtctatttttacgcattcaaatacgcgctagatatgcgcgttgagaatcgcgaggaagactacattcaatcacctgtgaaaaatgcctctggggaatttttttgttcaaaaaatgctggtaaaagcctcccacaagatagcgatgcaaacggtgcatataacattgccctgaaaggtattcttcaattacgcatgctgtctgagcagtacgaccccaacgcggaatctattagacttccgctgataaccaataaagcctggctgacattcatgcagtctggcatgaagacctggaaaaattagGAAATCATCCTTAGCGAAAGCTAAGGCTGATACTCTTCC

PC_Cpf1_5	CGTATGATAAATACTTCCTTCATATGCCTATTACGATCaattttaaatgttctgcaggaagcaaa<br>gtcaatgatatggttaatgctcatattcgagaggcaaaggatatgcatgtcattggaattgatcg<br>tggagaacgcaatctgctgtatatatgcgtgatagatagtcgagggacgattttggatcaaattt<br>ctctgaatacgattaacgatatagactatcatgatttattggagagtcgagacaaagaccgtcag<br>caggagcgccgaaactggcaaactatcgaagggatcaaggagctaaaacaaggctaccttagtca<br>ggcggttcatcggatagccgaactgatggtggcttataaggctgtagttgctttggaggatttga<br>atatggggttcaaacgtgggcggcagaaagtagaaagttctgtttatcagcagtttgagaaacag<br>ctgatagataagctcaactatcttgtggacaagaagaaaaggcctgaagatattggaggattgtt<br>gagagcctatcaatttacggccccatttaagagttttaaggaaatgggaaagcaaaacggcttct<br>tgttttatatcccggcttggaacacgagcaacatagatccgactactggatttgttaatttattt<br>catgcccagtatgaaaatgtagataaagcgaagagcttctttcaaaagtttgattcaattagtta<br>caacccgaagaaagactggtttgagtttgcattcgattataaaaactttactaaaaaggctgaag<br>gaagtcgttctatgtggatattatgcacacatggttcccgaataaagaattttagaaattcccag<br>aagaatggtcaatgggattccgaagaattcgccttgacggaggcttttaagtctctttttgtgcg<br>atatgagatagattataccgctgatttgaaaacagctattgtggacgaaaagcaaaaagacttct<br>tcgtggatcttctgaagctattcaaattgacagtacagatgcgcaacagctggaaagagaaggat<br>ttggattatctaatctctcctgtagcaggggctgatggccgtttcttcgatacaagagagggaaa<br>taaaagtctgcctaaggatgcagatgccaatggagcttataatattgccctaaaaggactttggg<br>ctctacgccagattcggcaaacttcagaaggcggtaaactcaaattggcgatttccaataaggaa<br>tggctacagtttgtgcaagagagatcttacgagaaagactgaGAAATCATCCTTAGCGAAAGCTA<br>AGGCTGATACTCTTCC
FB_Cpf1_5	CGTATGATAAATACTTCCTTCATATGCCTATTACGATCaacttcaaagctaccgggggtagctat<br>atcaaccaaacggtgttggaatacctgcagaataacccggaagtcaaaatcattgggctggaccg<br>cggagaacgtcaccttgtgtacttgaccttaatcgatcagcaaggcaacatcttaaaacaagaat<br>cgctgaataccattacggattcaaagattagcaccccgtatcataagctgctcgataacaaggag<br>aatgagcgcgacctggcccgtaaaaactggggcacggtggaaaacattaaggagttaaaggaggg<br>ttatatttcccaggtagtgcataagatcgccactctcatgctcgaggaaaatgcgatcgttgtca<br>tggaagacttaaacttcggatttaaacgtgggcgatttaaagtagagaaacaaatctaccagaag<br>ttagaaaaaatgctgattgacaaattaaattacttggtcctaaaagacaaacagccgcaagaatt<br>gggtggattatacaacgccctccaacttaccaataaattcgaaagttttcagaaaatgggtaaac<br>agtcaggctttctttttttatgttcctgcgtggaacacatccaaaatcgaccctacaaccggcttc<br>gtcaattacttctatactaaatatgaaaacgtcgacaaagcaaaagcattctttgaaaagttcga<br>agcaatacgttttaacgctgagaaaaaatatttcgagttcgaagtcaagaaatactcagacttta<br>accccaaagctgagggcacacagcaagcgtggacaatctgcacctacggcgagcgcatcgaaacg<br>aagcgtcaaaaagatcagaataacaaatttgtttcaacacctatcaacctgaccgagaagattga<br>agacttcttaggtaaaaatcagattgtttatggcgacggtaactgtataaaatctcaaatagcct<br>caaaggatgataaagcatttttcgaaacattattatattggttcaaaatgacactgcagatgcgc<br>aatagtgagacgcgtacagatattgattatcttatcagcccggtcatgaacgacaacggtacttt<br>ttacaactccagagactatgaaaaacttgagaatccaactctccccaaagatgctgatgcgaacg<br>gtgcttatcacatcgcgaaaaaaggtctgatgctgctgaacaaaatcgaccaagccgatctgact<br>aagaaagttgacctaagcatttcaaatcgggactggttacagtttgttcaaaagaacaaatgaGA<br>AATCATCCTTAGCGAAAGCTAAGGCTGATACTCTTCC

CR_Cpf1_5	CGTATGATAAATACTTCCTTCATATGCCTATTACGATCaaccgtaccaaaaacgactcttaccgt<br>ttcaacgcgcagatcaacaacttcctggcgaacaacaaagacatcaacatcatcggtgttgaccg<br>tggtgaaaaacacctggtttactactctgttatcacccaggcgtctgacatcctggaatctggtt<br>ctctgaacgaactgaacggtgttaactacgcggaaaaactgggtaaaaaagcggaaaaccgtgaa<br>caggcgcgtcgtgactggcaggacgttcagggtatcaaagacctgaaaaaaggttacatctctca<br>ggttgttcgtaaactggcggacctggcgatcaaacacaacgcgatcatcatcctggaagacctga<br>acatgcgtttcaaacaggttcgtggtggtatcgaaaaatctatctaccagcagctggaaaaagcg<br>ctgatcgacaaactgtctttcctggttgacaaaggtgaaaaaaacccggaacaggcgggtcacct<br>gctgaaagcgtaccagctgtctgcgccgttcgaaaccttccagaaaatgggtaaacagaccggta<br>tcatcttctacacccaggcgtcttacacctctaaatctgacccggttaccggttggcgtccgcac<br>ctgtacctgaaatacttctctgcgaaaaaagcgaaagacgacatcgcgaaattcaccaaaatcga<br>attcgttaacgaccgtttcgaactgacctacgacatcaaagacttccagcaggcgaaagaatacc<br>cgaacaaaaccgtttggaaagtttgctctaacgttgaacgtttccgttgggacaaaaacctgaac<br>cagaacaaaggtggttacacccactacaccaacatcaccgaaaacatccaggaactgttcaccaa<br>atacggtatcgacatcaccaaagacctgctgacccagatctctaccatcgacgaaaaacagaaca<br>cctctttcttccgtgacttcatcttctacttcaacctgatctgccagatccgtaacaccgacgac<br>tctgaaatcgcgaaaaaaaacggtaaagacgacttcatcctgtctccggttgaaccgttcttcga<br>ctctcgtaaagacaacggtaacaaactgccggaaaacggtgacgacaacggtgcgtacaacatcg<br>cgcgtaaaggtatcgttatcctgaacaaaatctctcagtactctgaaaaaaacgaaaactgcgaa<br>aaaatgaaatggggtgacctgtacgtttctaacatcgactgggacaacttcgtttgaGAAATCAT<br>CCTTAGCGAAAGCTAAGGCTGATACTCTTCC
SC_Cpf1_5	CGTATGATAAATACTTCCTTCATATGCCTATTACGATCaactaccaggcggcgaactctccgtct<br>aaattcaaccagcgtgttaacgcgtacctgaaagaacacccggaaaccccgatcatcggtatcga<br>ccgtggtgaacgtaacctgatctacatcaccgttatcgactctaccggtaaaatcctggaacagc<br>gttctctgaacaccatccagcagttcgactaccagaaaaaactggacaaccgtgaaaaagaacgt<br>gttgcggcgcgtcaggcgtggtctgttgttggtaccatcaaagacctgaaacagggttacctgtc<br>tcaggttatccacgaaatcgttgacctgatgatccactaccaggcggttgttgttctggaaaacc<br>tgaacttcggtttcaaatctaaacgtaccggtatcgcggaaaaagcggtttaccagcagttcgaa<br>aaaatgctgatcgacaaactgaactgcctggttctgaaagactacccggcggaaaaagttggtgg<br>tgttctgaacccgtaccagctgaccgaccagttcacctctttcgcgaaaatgggtacccagtctg<br>gtttcctgttctacgttccggcgccgtacacctctaaaatcgacccgctgaccggtttcgttgac<br>ccgttcgtttggaaaaccatcaaaaaccacgaatctcgtaaacacttcctggaaggtttcgactt<br>cctgcactacgacgttaaaaccggtgacttcatcctgcacttcaaaatgaaccgtaacctgtctt<br>tccagcgtggtctgccgggtttcatgccggcgtgggacatcgttttcgaaaaaaacgaaacccag<br>ttcgacgcgaaaggtaccccgttcatcgcgggtaaacgtatcgttccggttatcgaaaaccaccg<br>tttcaccggtcgttaccgtgacctgtacccggcgaacgaactgatcgcgctgctggaagaaaaag<br>gtatcgttttccgtgacggttctaacatcctgccgaaactgctggaaaacgacgactctcacgcg<br>atcgacaccatggttgcgctgatccgttctgttctgcagatgcgtaactctaacgcggcgaccgg<br>tgaagactacatcaactctccggttcgtgacctgaacggtgtttgcttcgactctcgtttccaga<br>acccggaatggccgatggacgcggacgcgaacggtgcgtaccacatcgcgctgaaaggtcagctg<br>ctgctgaaccacctgaaagaatctaaagacctgaaactgcagaacggtatctctaaccaggactg<br>gctggcgtacatccaggaactgcgtaactatgaGAAATCATCCTTAGCGAAAGCTAAGGCTGATA<br>CTCTTCC

SD_Cpf1_6	CGGCTGCATTTTTTATGTGCCTGCTGCATACACGAGCaagattgatccgaccacgggcttcgccaatgttctgaatctgtcgaaggtacgcaatgttgatgcgatcaaaagctttttttctaacttcaacgaaattagttatagcaagaaagaagcccttttcaaattctcattcgatctggattcactgagtaagaaaggctttagtagctttgtgaaatttagtaagagtaaatggaacgtctacacctttggagaacgtatcataaagccaaagaataagcaaggttatcgggaggacaaaagaatcaacttgaccttcgagatgaagaagttacttaacgagtataaggtttcttttgatcttgaaaataacttgattccgaatctcacgagtgccaacctgaaggatacttttggaaagagctattctttatcttcaagactacgctgcagctccgtaacagcgttactaacggtaaagaagatgtgctcatctctccggtcaaaaatgcgaagggtgaattcttcgtttcgggaacgcataacaagactcttccgcaagattgcgatgcgaacggtgcataccatattgcgttgaaaggtctgatgatactcgaacgtaacaaccttgtacgtgaggagaaagatacgaaaaagattatggcgatttcaaacgtggattggttcgagtacgtgcagaaacgtagaggcgttctgtaaGAAATCATCCTTAGCGAAAGCTAAGGCTGATACTCTTCC
CT_Cpf1_6	CGGCTGCATTTTTTATGTGCCTGCTGCATACACGAGCaaaatcgacccgaccaccggtttcgttaacctgttcaacacctcttctaaaaccaacgcgcaggaacgtaaagaattcctgcagaaattcgaatctatctcttactctgcgaaagacggtggtatcttcgcgttcgcgttcgactaccgtaaattcggtacctctaaaaccgaccacaaaaacgtttggaccgcgtacaccaacggtgaacgtatgcgttacatcaaagaaaaaaaacgtaacgaactgttcgacccgtctaaagaaatcaaagaagcgctgacctcttctggtatcaaatacgacggtggtcagaacatcctgccggacatcctgcgttctaacaacaacggtctgatctacaccatgtactcttctttcatcgcggcgatccagatgcgtgtttacgacggtaaagaagactacatcatctctccgatcaaaaactctaaaggtgaattcttccgtaccgacccgaaacgtcgtgaactgccgatcgacgcggacgcgaacggtgcgtacaacatcgcgctgcgtggtgaactgaccatgcgtgcgatcgcggaaaaattcgacccggactctgaaaaaatggcgaaactggaactgaaacacaaagactggttcgaattcatgcagacccgtggtgactaaGAAATCATCCTTAGCGAAAGCTAAGGCTGATACTCTTCC
TX_Cpf1_6	CGGCTGCATTTTTTATGTGCCTGCTGCATACACGAGCttctgttttacgtgccggcagattatacttcaaaaatcgatccaacaactggctttgtgaacttcctggacctgagatatcagtctgtagaaaaagctaaacaacttcttagcgattttaatgccattcgttttaacagcgttcagaattactttgaattcgaaattgactataaaaaacttactccgaaacgtaaagtcggaacccaaagtaaatgggtaatttgtacgtatggcgatgtcaggtatcagaaccgtcggaatcaaaaaggtcattgggagaccgaagaagtgaacgtgaccgaaaagctgaaggctctgttcgccagcgattcaaaaactacaactgtgatcgattacgcaaatgatgataacctgatagatgtgattttagagcaggataaagccagctttttttaaagaactgttgtggctcctgaaacttacgatgaccttacgacattccaagatcaaatcggaagatgattttattctgtcaccggtcaagaatgagcagggtgaattctatgatagtaggaaagccggcgaagtgtggccgaaagacgccgacgccaatggcgcctatcatatcgcgctcaaagggctttggaatttgcagcagattaaccagtgggaaaaaggtaaaaccctgaatctggctatcaaaaaccaggattggtttagctttatccaagagaaaccgtatcaggaatgaGAAATCATCCTTAGCGAAAGCTAAGGCTGATACTCTTCC

CA_Cpf1_6	CGGCTGCATTTTTTATGTGCCTGCTGCATACACGAGCggttatcttttatataccggcagcgttc<br>actagtaaaatagatccgaccactggtttcgccgatctctttgccctgagtaacgttaaaaacgt<br>agcgagcatgcgtgaattcttttccaaaatgaaatctgtcatttatgataaagctgaaggcaaat<br>tcgcattcacctttgattacttggattacaacgtgaagagcgaatgtggtcgtacgctgtggacc<br>gtttacaccgttggtgagcgcttcacctattcccgtgtgaaccgcgaatatgtacgtaaagtccc<br>caccgatattatctatgatgccctccagaaagcaggcattagcgtcgaaggagacttaagggaca<br>gaattgccgaaagcgatggcgatacgctgaagtctatttttttacgcattcaaatacgcgctagat<br>atgcgcgttgagaatcgcgaggaagactacattcaatcacctgtgaaaaatgcctctggggaatt<br>tttttgttcaaaaaatgctggtaaaagcctcccacaagatagcgatgcaaacggtgcatataaca<br>ttgccctgaaaggtattcttcaattacgcatgctgtctgagcagtacgaccccaacgcggaatct<br>attagacttccgctgataaccaataaagcctggctgacattcatgcagtctggcatgaagacctg<br>gaaaaattagGAAATCATCCTTAGCGAAAGCTAAGGCTGATACTCTTCC
PC_Cpf1_6	CGGCTGCATTTTTTATGTGCCTGCTGCATACACGAGCgttttatatcccggcttggaacacgagc<br>aacatagatccgactactggatttgttaatttatttcatgcccagtatgaaaatgtagataaagc<br>gaagagcttctttcaaaagtttgattcaattagttacaacccgaagaaagactggtttgagtttg<br>cattcgattataaaaactttactaaaaaggctgaaggaagtcgttctatgtggatattatgcaca<br>catggttcccgaataaagaattttagaaattcccagaagaatggtcaatgggattccgaagaatt<br>cgccttgacggaggcttttaagtctctttttgtgcgatatgagatagattataccgctgatttga<br>aaacagctattgtggacgaaaagcaaaaagacttcttcgtggatcttctgaagctattcaaattg<br>acagtacagatgcgcaacagctggaaagagaaggatttggattatctaatctctcctgtagcagg<br>ggctgatggccgtttcttcgatacaagagagggaaataaaagtctgcctaaggatgcagatgcca<br>atggagcttataatattgccctaaaaggactttgggctctacgccagattcggcaaacttcagaa<br>ggcggtaaactcaaattggcgatttccaataaggaatggctacagtttgtgcaagagagatctta<br>cgagaaagactgaGAAATCATCCTTAGCGAAAGCTAAGGCTGATACTCTTCC
FB_Cpf1_6	CGGCTGCATTTTTTATGTGCCTGCTGCATACACGAGCttctttttatgttcctgcgtggaacac<br>atccaaaatcgaccctacaaccggcttcgtcaattacttctatactaaatatgaaaacgtcgaca<br>aagcaaaagcattctttgaaaagttcgaagcaatacgttttaacgctgagaaaaaatatttcgag<br>ttcgaagtcaagaaatactcagactttaaccccaaagctgagggcacacagcaagcgtggacaat<br>ctgcacctacggcgagcgcatcgaaacgaagcgtcaaaaagatcagaataacaaatttgtttcaa<br>cacctatcaacctgaccgagaagattgaagacttcttaggtaaaaatcagattgtttatggcgac<br>ggtaactgtataaaatctcaaatagcctcaaaggatgataaagcatttttcgaaacattattata<br>ttggttcaaaatgacactgcagatgcgcaatagtgagacgcgtacagatattgattatcttatca<br>gcccggtcatgaacgacaacggtactttttacaactccagagactatgaaaaacttgagaatcca<br>actctccccaaagatgctgatgcgaacggtgcttatcacatcgcgaaaaaaggtctgatgctgct<br>gaacaaaatcgaccaagccgatctgactaagaaagttgacctaagcatttcaaatcgggactggt<br>tacagtttgttcaaaagaacaaatgaGAAATCATCCTTAGCGAAAGCTAAGGCTGATACTCTTCC

CR_Cpf1_6	CGGCTGCATTTTTTATGTGCCTGCTGCATACACGAGCtctacacccaggcgtcttacacctctaa<br>atctgacccggttaccggttggcgtccgcacctgtacctgaaatacttctctgcgaaaaaagcga<br>aagacgacatcgcgaaattcaccaaaatcgaattcgttaacgaccgtttcgaactgacctacgac<br>atcaaagacttccagcaggcgaaagaatacccgaacaaaaccgtttggaaagtttgctctaacgt<br>tgaacgtttccgttgggacaaaaacctgaaccagaacaaaggtggttacacccactacaccaaca<br>tcaccgaaaacatccaggaactgttcaccaaatacggtatcgacatcaccaaagacctgctgacc<br>cagatctctaccatcgacgaaaaacagaacacctctttcttccgtgacttcatcttctacttcaa<br>cctgatctgccagatccgtaacaccgacgactctgaaatcgcgaaaaaaaacggtaaagacgact<br>tcatcctgtctccggttgaaccgttcttcgactctcgtaaagacaacggtaacaaactgccggaa<br>aacggtgacgacaacggtgcgtacaacatcgcgcgtaaaggtatcgttatcctgaacaaaatctc<br>tcagtactctgaaaaaaacgaaaactgcgaaaaaatgaaatggggtgacctgtacgtttctaaca<br>tcgactgggacaacttcgtttgaGAAATCATCCTTAGCGAAAGCTAAGGCTGATACTCTTCC
SC_Cpf1_6	CGGCTGCATTTTTTATGTGCCTGCTGCATACACGAGCcgttccggcgccgtacacctctaaaatc<br>gacccgctgaccggtttcgttgacccgttcgtttggaaaaccatcaaaaaccacgaatctcgtaa<br>acacttcctggaaggtttcgacttcctgcactacgacgttaaaaccggtgacttcatcctgcact<br>tcaaaatgaaccgtaacctgtctttccagcgtggtctgccgggtttcatgccggcgtgggacatc<br>gttttcgaaaaaaacgaaacccagttcgacgcgaaaggtaccccgttcatcgcgggtaaacgtat<br>cgttccggttatcgaaaaccaccgtttcaccggtcgttaccgtgacctgtacccggcgaacgaac<br>tgatcgcgctgctggaagaaaaaggtatcgttttccgtgacggttctaacatcctgccgaaactg<br>ctggaaaacgacgactctcacgcgatcgacaccatggttgcgctgatccgttctgttctgcagat<br>gcgtaactctaacgcggcgaccggtgaagactacatcaactctccggttcgtgacctgaacggtg<br>tttgcttcgactctcgtttccagaacccggaatggccgatggacgcggacgcgaacggtgcgtac<br>cacatcgcgctgaaaggtcagctgctgctgaaccacctgaaagaatctaaagacctgaaactgca<br>gaacggtatctctaaccaggactggctggcgtacatccaggaactgcgtaactatgaGAAATCAT<br>CCTTAGCGAAAGCTAAGGCTGATACTCTTCC
lacZ-1	TCCTCTGGCGGAAAGCCTACACGAAGCGATTTTCTTTATGGCAGGGTGAAACGCAGGTCGCCAGC<br>GGCACCGCGCCTTTCTAATAAGAAATTATCGATGAGCGTGGTGGTTATGCCGATCGCGTCACACT<br>ACGTCTTTGACAGCTAGCTCAGTCCTAGGTATAATACTAGTGGAATTTCTACTCTTGTAGATGGC<br>GGTGAAATTATCGATGAATCCCAGAAAAGACCCGTCCG
lacZ-2	TCCTCTGGCGGAAAGCCTACACGAAGCGATGTGGTACACGCTGTGCGACCGCTACGGCCTGTATG<br>TGGTGGATGAAGCCTAATAAGAGACCCACGGCATGGTGCCAATGAATCGTCTGACCGATGATCCG<br>CGCTGGCTATTGACAGCTAGCTCAGTCCTAGGTATAATACTAGTGGAATTTCTACTCTTGTAGAT<br>AATATTGGCTTCATCCACCAATCCCAGAAAAGACCCGTCCG
lacZ-3	TCCTCTGGCGGAAAGCCTACACGAAGCGATGCGAATTCCACGATGCTGATGCGCAGAACTCTCAC<br>AGCTATTGCCGCGAAATTCTGGAGCGGCGGTAATTTTGTATAGAATTTACGGCTAGCGCTTGACA<br>GCTAGCTCAGTCCTAGGTATAATACTAGTGGAATTTCTACTCTTGTAGATATCAACATTAAATGT<br>GAGCGATCCCAGAAAAGACCCGTCCG
Galk-1	TCCTCTGGCGGAAAGCCTGTGAACGAGCATCCAAAGGTGTGGCTGTCGTCATCATCAACAGTAAC<br>TTCAAACGTACCCTGGTTGGCAGCGAATACAACACCTAGGGTGAACAGTGCGAGACCGGTGCGCG<br>TTTCTTCCAGCAGCCAGCCCTGCGTGATGTCATTGACAGCTAGCTCAGTCCTAGGTATAATACTA<br>GTGGAATTTCTACTCTTGTAGATGCACTGTTCACGACGGGTGTATCCCAGAAAAGACCCGTCCGC<br>CATGCCGTAGCACTGTGACCCGGCCAACTCCCACCATTTG
Galk-2	TTCCAGCTCGAAGGCGATCGTGCACAATATGCTGGCTGCTGGAAGAAACGCGCACCGGTTTCGCA<br>CTGTTCACCCTAGGTGTTGTATTCGCTGCCAACCAGGGTACGCTTAAAGTTACTGTTGATGATGA<br>CGACAGCCACACCTTTGGGCTTGACAGCTAGCTCAGTCCTAGGTATAATACTAGTGGAATTTCTA<br>CTCTTGTAGATAAACGTACCCTGGTTGGCAGATCCCAGAAAAGACCCGTCCGCCATGCCGTACCG<br>ACCCCAATACCCGATTCCGACACCGTAGCACTGTGACCCG

Galk-3	TTCCAGCTCGAAGGCGATCTAATGCTCACTATCGCGTGGTGCACAACTGATCACGGTTTGATAATCAATCGCGCAGGGCAGAACGAAACCGTCGTTGTAGTCGGTGTGTTCACCAATCAAATTCACGCGGCCAGGTTATTAAATGGTGTGAGTGGCAGGGTAACCGAATGCGTTGGCAAACAGAGATTGTGTTTTTTCTTTCAGACTCATTTCTTACACTCCGGATTCGCGAAAATGGATATCGCTGACTGCGCGCAAACGTTGACAGCTAGCTCAGTCCTAGGTATAATACTAGTGTCAAAAGACCTTTTTAATTTCTACTCTTGTAGATGCTACCCTGCCACTCACACCATCCCAGAAAAGACCCGTCCG
CAN1	GTTCGAAACTTCTCCGCAGTGAAAGATAAATGATCACGTTCTCTATGGAGGATGGCATAGGTGATGAAGATGAAGGAGAAGTACAGAACGCTGAAGTGAAGAGAGAGCTTGACATATTGGTATGATTGCCCTTGGTGGTACTATTGGTACAGGTCTTTTCATTGGTTTATCCACACCTCTGACCAACGCCGGATCGTCAAAAGACCTTTTTAATTTCTACTCTTGTAGATCTTAAGCTCTCTCTTCACTTCTGCCCGCTTTCCACCGGTGGTCTCTAGAGCTATGCTG
spacer for DNMT1	CTGATGGTCCATGTCTGTTA
