# Supplementary material for: Synthetic chimeric nucleases function for efficient genome editing
Source: Nat Commun. 2019 Dec 4;10:5524. doi: 10.1038/s41467-019-13500-y (PMC6892893; doi:10.1038/s41467-019-13500-y)
Supplement: Supplementary file 5 — Description of Additional Supplementary Files [file 41467_2019_13500_MOESM5_ESM.pdf]

Title: Supplementary Data 1
Description: The oligos and sequences used in this study
